# AI-Driven Breast Cancer Diagnosis: A Systematic Review of Imaging Modalities, Deep Learning, and Explainability

**DOI:** 10.3390/cancers18081305

**Published:** 2026-04-20

**Authors:** Margo Sabry, Hossam Magdy Balaha, Khadiga M. Ali, Ali Mahmoud, Dibson Gondim, Mohammed Ghazal, Tayseer Hassan A. Soliman, Ayman El-Baz

**Affiliations:** 1 Information Systems Department, Assiut University, Assiut 71515, Egypt; margo.sabry@aun.edu.eg (M.S.); taysirhs@aun.edu.eg (T.H.A.S.); 2 Bioengineering Department, J.B. Speed School of Engineering, University of Louisville, Louisville, KY 40292, USA; hmbala01@louisville.edu (H.M.B.); ali.mahmoud@louisville.edu (A.M.); 3 Pathology Department, Faculty of Medicine, Mansoura University, Mansoura 35516, Egypt; kh.abdelrahman@mans.edu.eg; 4 Department of Pathology and Laboratory Medicine, University of Louisville, Louisville, KY 40292, USA; dibson.gondim@louisville.edu; 5 Electrical, Computer, and Biomedical Engineering Department, Abu Dhabi University, Abu Dhabi 59911, United Arab Emirates; mohammed.ghazal@adu.ac.ae; 6 Research Institute for AI and Emerging Technology, Liwa University, Abu Dhabi 41009, United Arab Emirates

**Keywords:** breast cancer (BC), computer-aided diagnosis (CAD), deep learning (DL), eXplainable artificial intelligence (XAI), machine learning (ML)

## Abstract

**Background**: This article provides a comprehensive overview of recent advancements in artificial intelligence (AI) and deep-learning technologies for breast cancer (BC) diagnosis across various imaging modalities. **Methods**: A systematic review was conducted in strict adherence to the PRISMA guidelines, incorporating a comparative analysis of 65 peer-reviewed studies published between 2018 and 2024. The evaluation focused on diagnostic performance, architectural developments, and clinical integration strategies. **Results**: The review synthesizes primary findings on convolutional neural networks (CNNs), emerging architectures including graph neural networks, and hybrid models, with diagnostic accuracy, risk prediction, and personalized screening strategies identified as the leading research domains. Notable achievements include CNNs attaining up to 98.5% accuracy in mammography and Vision Transformers reaching 96% in histopathological analysis. Furthermore, the implementation of explainable AI methodologies, such as SHAP, LIME, and Grad-CAM, is emphasized for maintaining transparency, trust, and accountability in clinical decision-making. **Conclusions**: AI constitutes a pivotal factor in facilitating early BC diagnosis and optimizing treatment outcomes. Nevertheless, significant challenges persist, including dataset heterogeneity, model generalizability, standardization of imaging protocols, computational resource limitations, and the seamless integration of these technologies into established clinical workflows. Future research must prioritize robust multi-dataset validation and standardized implementation frameworks to overcome existing limitations and advance successful BC diagnostic practices.

## Simple Summary

Breast cancer remains a leading cause of cancer-related mortality among women worldwide. Early and accurate diagnosis significantly improves patient outcomes. This systematic review examines how artificial intelligence (AI) and deep-learning technologies are transforming breast cancer diagnosis across multiple imaging modalities, including mammography, ultrasound, MRI, molecular breast imaging, PET, and histopathology. We analyzed 65 peer-reviewed studies published between 2018 and 2024, focusing on convolutional neural networks, vision transformers, graph neural networks, and explainable AI methods. Our findings indicate that AI models can achieve diagnostic accuracies exceeding 96% in certain contexts, supporting radiologists in detecting subtle abnormalities and reducing false positives. However, challenges remain regarding dataset standardization, model generalizability, and clinical integration. We emphasize the importance of explainable AI techniques to foster clinician trust and highlight future directions for translating these innovations into routine clinical practice.

## 1. Introduction

Breast cancer (BC) continues to be one of the most common and lethal cancers among women globally. Lowering mortality and increasing the survival of patients relies greatly on prompt diagnosis and accurate prognosis. In recent years, major developments in imaging techniques and AI have changed the landscape of BC diagnosis such that we can achieve more accurate, rapid, and personalized diagnostic tools [1].

Mammography and ultrasound remain the basis for the screening of BC using conventional imaging methods. Mammography, through the use of low-dose X-rays, is a powerful technique to detect small tumors at an early stage, and it has progressed with contrast-enhanced mammography (CEM), digital breast tomosynthesis (DBT), and digital mammography (DM) to increase its diagnostic precision with a better image quality, as well as 3D views [2]. Ultrasound is particularly useful for dense breast tissue, provides real-time imaging, and advanced techniques such as elastography, which enhances lesion characterization [3].

Magnetic resonance imaging (MRI) is essential for identifying invasive tumors, evaluating the effectiveness of treatment, and performing high-resolution and thorough tissue characterization, combining diffusion-weighted imaging (DWI) and dynamic contrast-enhanced magnetic resonance imaging (DCE-MRI), enabling personalized medicine by identifying molecular subtypes [4]. Molecular breast imaging (MBI) and positron emission tomography (PET) highlight metabolic activity, allowing early detection, especially in dense tissue [5]. Histopathology, the gold standard for definitive diagnosis, benefits from digital pathology and AI, enhancing precision and reducing variability among pathologists by identifying subtle patterns and biomarkers [6].

Nonetheless, a few technical and medical challenges are still present when talking about standardization and large-scale clinical usage of the method. Differences in imaging protocols, types of equipment, and the requirement for AI to be integrated in the clinical-pathway are some of the issues that have to be resolved by subsequent research and development [7].

The novelty of this survey is multifaceted, extending beyond current limitations to address critical challenges in BC diagnosis. This publication is a review from 2018 to 2024 of all the advances in different imaging modalities, AI technologies, and their impact on the diagnostic accuracy and efficiency of imaging. This review, through the lens of various imaging modalities such as mammography, ultrasound, MRI, MBI, and PET, combined with AI-enabled diagnosis and explainability approaches, highlights how these technologies empower early detection, favorable patient outcomes, and tailored medicine. The article also examines the difficulties in standardizing imaging protocols, the need for specially designed equipment, and the integration of AI into the clinical workflow. Acknowledging these factors, this survey aims to serve as a strategic instrument for shaping research and development directions, such as overcoming the current limitations and being able to improve the existing BC diagnostic protocols.

The rest of this survey is organized as follows: Section 2 reviews various BC diagnosis modalities; Section 3 discusses BC datasets; Section 4 demonstrates the diagnostic and explainability framework; and Section 5 presents the related AI-based systems; Section 6 presents the limitations of current research; and finally Section 7 concludes the manuscript and suggests future directions.

## 2. Breast Cancer Diagnosing Modalities

**Figure 1:**
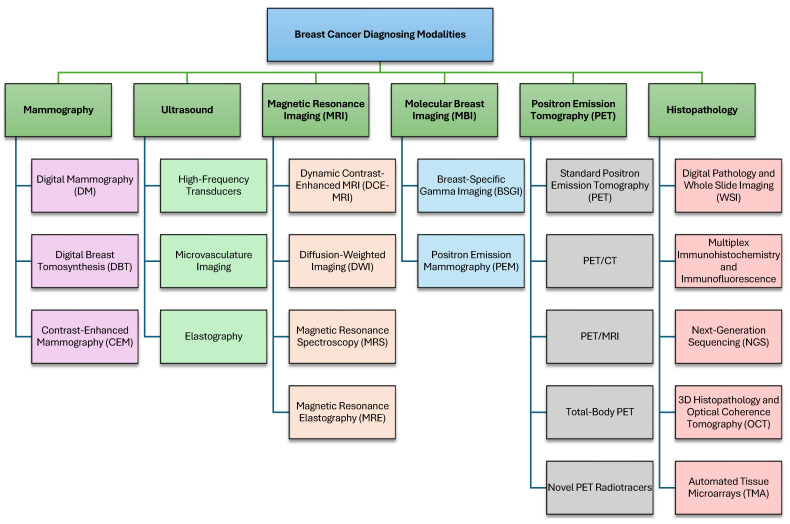
A comprehensive overview of various diagnostic techniques/modalities used for BC detection.

Over the past few years, BC diagnosis has notably advanced in terms of imaging modality and AI integration, which have been critical in increasing detection and diagnosing accuracy [8,9]. Conventional imaging modalities, such as mammography and ultrasound, still continue to serve as the cornerstone in detecting BC, but more recent modalities, including digital breast tomosynthesis (DBT), contrast-enhanced mammography (CEM), magnetic resonance imaging (MRI), and molecular breast imaging (MBI), have increased diagnostic potential [5]. These methods provide better resolution and tissue contrast, which is essential for treatment planning and early diagnosis. Figure 1 gives a detailed summary of different BC diagnostic methods/modalities. These modalities are described in the following subsections.

### 2.1. Mammography

Mammography is a breast imaging modality that generates high-resolution radiographic images using low-dose ionizing radiation [10]. The first and foremost aim of mammography is to help diagnose BC early, sometimes even before signs and symptoms develop, to detect these changes in the tissue of the breast [11]. It requires flattening the breast between two plates in order to distribute the tissue evenly so that clear images can be captured. These images are subsequently evaluated for any evidence of cancer, including lumps, calcifications, or other abnormal changes [12,13].

There is a substantial benefit of mammography in terms of early diagnosis and treatment of BC. It is through mammography that tumors too small to be felt can be identified, allowing an early diagnosis and treatment, thus improving the overall survival rates [14]. It has been found to reduce BC mortality [15]. In addition, technological developments such as digital mammography, tomosynthesis, and AI have only increased both diagnostic accuracy and workflow efficiency. Advanced mammography is widely used for BC diagnosis, including several techniques:

- Digital Mammography (DM) uses computer-aided detection, replacing conventional film with electronic devices that capture images of the breast, which are stored directly in a computer. This development offers improved aesthetic quality, enhanced image handling, and better storage and sharing capabilities for second opinions and remote consultations. It has been scientifically proven that digital mammography increases diagnostic accuracy, particularly among women with dense breasts [10,16].
- Digital Breast Tomosynthesis (DBT), also known as 3D mammography, is an emerging imaging add-on technique to take multiple X-ray images of the breast from a set of angles [17]. Subsequently, these images are reconstructed into a 3D volume, providing a more comprehensive visualization of the breast tissue, as opposed to traditional 2D mammography. This approach improves human BC detection and decreases false-positive diagnoses by providing a clearer and more detailed inside visualization of the breast, compared with standard mammography, which enables the radiologists to better recognize the abnormalities [2].
- Contrast-Enhanced Mammography (CEM) is performed by injecting a contrast in the bloodstream before mammography. The contrast regions with higher blood volume that are often associated with malignant lesions, and thus increase detection rates, especially in women with dense breasts or when standard mammography is inconclusive [18].

**Figure 2:**
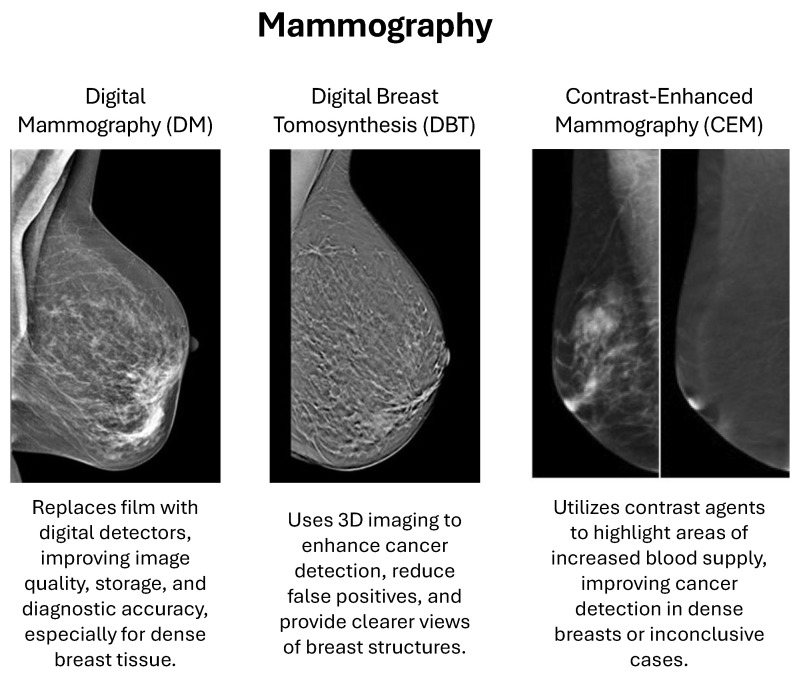
Graphical visualization of the three techniques in the mammography modality: DM, DBT, and CEM [19,20].

Figure 2 provides a graphical visualization of the three techniques in the mammography modality: DM, DBT, and CEM [19,20].

### 2.2. Ultrasound

Ultrasound is an important diagnostic method in BC diagnosis and therapy, especially to study the lesions identified in mammography or in women with dense breasts. Ultrasounds use high-frequency sound waves instead of X-rays to produce detailed images of breast tissue, as opposed to mammography. This modality is particularly suitable for differentiating solid masses from cysts and is also useful in guiding needle biopsies [3,21]. Recent developments, including high-frequency transducers, microvasculature imaging, elastography, and contrast-enhanced ultrasound, have greatly enhanced the diagnostic performance.

There has been a significant increase in the spatial resolution and tissue characterization of ultrasound images with the advancement of technologies. Higher frequency transducers afford better differentiation between low grayscale tones and features of lesions, while microvasculature imaging and elastography allow measurement of blood flow and tissue stiffness, respectively [22]. Contrast-enhanced ultrasound also helps to better visualize tumor vasculature. This is important for accurate diagnosis and to formulate treatment strategies. Ultrasound is non-ionizing radiation, which allows it to be used repeatedly and also to transmit images in a compact, relatively inexpensive package for the benefit of clinical treatment. There are a variety of advanced forms of ultrasound utilized for BC diagnosis, including:

- High-Frequency Transducers work at frequencies typically greater than 15 MHz, giving images with detailed superficial structures due to higher spatial resolution. The increased resolution results in more visualization of fine anatomical details, improving diagnostic sensitivity for small lesions and structural abnormalities [23,24].
- Microvasculature Imaging visualizes tiny blood vessels within tissues using advanced Doppler technology to capture slow and small blood flow. It is critical in oncology for identifying tumor angiogenesis and is used in assessing microcirculation in organs and tissues. It provides insights into vascular structures and blood flow patterns, improving the capacity to distinguish between vascularity-based benign and malignant tumors [24].
- Elastography evaluates tissue stiffness by measuring the response to an external force and has two major methods: strain elastography and shear-wave elastography [25]. It allows non-invasive assessment of tissue elasticity and fibrosis, enhancing the diagnostic confidence with the additional data concerning the composition of tissue [26].

**Figure 3:**
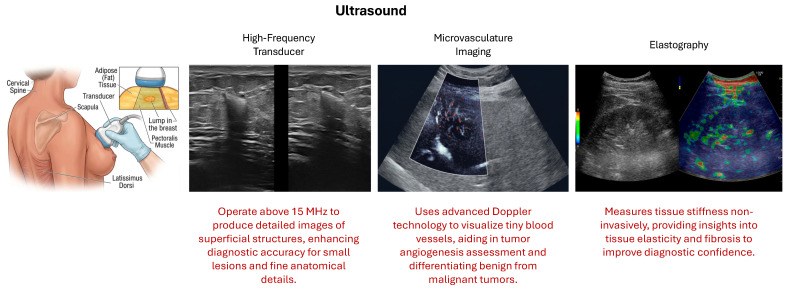
Graphical visualization of the three techniques in the ultrasound modality [27,28,29].

Figure 3 provides a graphical visualization of the three techniques in the ultrasound modality [27,28,29].

### 2.3. Magnetic Resonance Imaging (MRI)

Magnetic resonance imaging (MRI) excels in providing high-contrast images of soft tissues, making it particularly effective in detecting and characterizing breast lesions that might be missed by other modalities such as mammography and ultrasound. This makes it especially valuable for screening high-risk populations, such as women with a familial predisposition to BC or those presenting with dense breast tissue, where traditional methods might be less effective [24].

Techniques like diffusion-weighted imaging (DWI) and dynamic contrast-enhanced magnetic resonance imaging (DCE-MRI) enhance diagnostic accuracy by providing detailed insights into the vascular properties and cellular integrity of lesions. In addition, MRI functional imaging is valuable for assessing tumor biology and the prediction of treatment responses, aiding personalized treatment plans [30]. There are several advanced types of MRI used for BC diagnosis, including:

- Dynamic Contrast-Enhanced MRI (DCE-MRI) involves the use of contrast agents to enhance the visualization of blood flow and vascular properties within breast tissues. This technique helps in distinguishing benign from malignant lesions by analyzing how these tissues uptake and wash out the contrast agent. Studies have shown that DCE-MRI can detect BCs with a sensitivity of up to 87%, significantly higher than mammography. DCE-MRI’s ability to assess tumor angiogenesis and microvascular density makes it a crucial tool in evaluating tumor aggressiveness and planning treatment strategies. Dong et al. [31] demonstrated that DCE-MRI has a pooled diagnostic sensitivity of 0.87 and specificity of 0.74, respectively, with a diagnostic odds ratio (DOR) of 18.83, highlighting its effectiveness in BC detection.
- Diffusion-Weighted Imaging (DWI) measures the flow of water molecules through tissues and indicates information on tissue cellularity and structural integrity. It is of particular value in malignancy detection because lesions are more likely to have restricted diffusion in the malignancy relative to benign lesions. DWI improves the diagnostic performance of MRI by differentiating high cellular density, which is usually malignant. This method is important for early discovery and evaluation of the effect of treatment, since changes in tissue diffusivity may reflect therapeutic effects. Rodriguez-Soto et al. [32] also reported that advanced DWI techniques, such as restriction spectrum imaging (RSI), improved the tumor conspicuity significantly when compared with conventional methods.
- Magnetic Resonance Spectroscopy (MRS) examines the chemical content of breast tissue, providing more specific biochemical information, which is a valuable adjunct to conventional MRI images. MRS can detect particular metabolites related to malignant transformation, for example, heightened choline levels being a signal of an increase in cellular proliferation [33]. This subtype improves the specificity in differentiating various breast lesions and also provides additional information suggestive of the diagnosis and management of BC. MRS has been shown to help improve the accuracy of diagnosis and characterization of BC when combined with other MRI techniques [34].
- Magnetic Resonance Elastography (MRE) is capable of evaluating the mechanical properties of breast tissues, with tissue stiffness playing an important role. The stiffness of malignant tumors is usually higher than that of benign lesions because of cellularity and fibrosis [35]. MRE allows for a non-invasive assessment of these mechanical differences, which can help differentiate benign from malignant tumors and may be used to enhance BC identification and characterization in challenging clinical situations [36].

**Figure 4:**
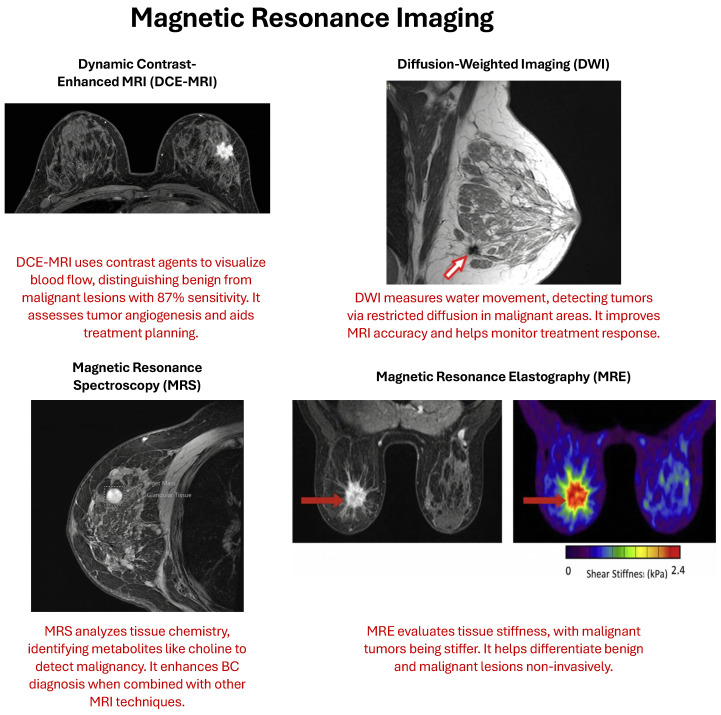
Graphical visualization of the four techniques in the MRI modality: DCE-MRI, DWI, MRS, and MRE.

Figure 4 provides a graphical visualization of the four techniques in the MRI modality [37,38,39,40].

### 2.4. Molecular Breast Imaging (MBI)

MBI is an advanced nuclear medicine method for detecting BC. It involves the use of a radiotracer, typically Technetium-99m sestamibi, which is injected into the bloodstream and absorbed by cancer cells as a result of their increased metabolic activity. The radiotracer emits gamma rays, which are captured by a specialized gamma camera to produce in-depth images of the breast tissue. MBI is particularly beneficial for women with dense breast tissue, where traditional mammography may have reduced sensitivity [41].

One of the primary strengths of MBI is its ability to detect cancers that may be missed by mammography, especially in dense breast tissue. MBI has demonstrated a higher sensitivity for detecting small and otherwise occult BCs, with studies showing incremental cancer detection rates of up to 9.3 per 1000 exams when used alongside mammography. Additionally, MBI offers a high negative predictive value and is less affected by breast density, making it a reliable supplementary screening tool. It also provides functional imaging, which can help in assessing the response to neoadjuvant therapy and evaluating the extent of disease [42].

MBI encompasses several advanced imaging techniques, primarily including breast-specific gamma imaging (BSGI) and positron emission mammography (PEM) [43]:

- Breast-specific gamma imaging (BSGI) utilizes a gamma camera to detect gamma rays emitted by a radiotracer, commonly Technetium-99m sestamibi, injected into the patient. Cancer cells, due to their higher metabolic activity, absorb more of the radiotracer, allowing for the creation of high-resolution images that highlight areas of concern. BSGI is particularly effective in dense breast tissue where mammography might be less sensitive [44].
- Positron emission mammography (PEM) involves the use of a PET scanner to detect positrons emitted by a radiotracer such as Fluorodeoxyglucose (FDG). This method highlights areas with elevated glucose metabolism, a characteristic feature of cancer cells. PEM provides high-resolution images and is useful for identifying and characterizing breast lesions, especially in dense breast tissue [45].

MBI has some notable limitations despite its benefits. A major concern is the radiation exposure required, although recent advancements have reduced the administered dose. In addition, MBI is not available to many recipients because it requires special equipment and nuclear medicine facilities, which are not as easily available in some regions [46]. The procedure also involves longer imaging times compared to traditional mammography, posing logistical challenges for both patients and healthcare providers. Additionally, MBI may have trouble clearly displaying lesions close to the axillary lymph nodes and chest wall due to limitations in the detector’s field of view [47].

### 2.5. Positron Emission Tomography (PET)

PET allows clinicians to visualize and measure metabolic processes within the body using radiotracers such as FDG. The radiotracer builds up in tissues with high metabolic activity, including cancer cells, after being injected into the patient’s circulation. The PET scanner detects the gamma rays emitted by the radiotracer, creating detailed images that reflect the functional activity of organs and tissues [48].

One of the primary strengths of PET is its ability to detect abnormalities at the molecular level, often before structural changes become apparent on other imaging modalities like CT or MRI. This high sensitivity makes PET particularly valuable in oncology for early cancer detection, staging, and monitoring treatment response. In addition, in a single session, PET can be used in conjunction with CT or magnetic resonance imaging to offer anatomical and metabolic information, improving diagnostic accuracy. PET/CT, specifically using 18F-FDG, is instrumental in identifying malignant lesions, assessing the extent of disease spread, and evaluating the effectiveness of treatment [49]. PET has seen several recent technological advancements:

- Standard PET is the most commonly used imaging tool for assessing the body’s metabolic activity in a clinical setting [50].
- PET/CT combines the functional imaging power of PET with the anatomical detail of CT. This imaging modality, as a hybrid system, allows the precise location of metabolism-related anomalies within the body, improving diagnostic accuracy. In oncology, PET/CT is especially useful for cancer staging, monitoring a patient’s response to therapy, and identifying recurrent disease [51].
- PET/MRI overlays high spatial resolution anatomic details imparted by MRI on the metabolic information available from PET, thus it can potentially be considered to be an ideal imaging modality for soft tissues. This combination is particularly advantageous for neurology and oncology imaging, where high-quality soft tissue contrast is paramount [50].
- Total-body PET advanced imaging modality enables imaging the whole body simultaneously. This may be of great utility in the study of systemic diseases, as well as for dynamic imaging of physiological processes. Total-body PET can be used to provide a complete picture of disease spread, to assess response to therapy, and to monitor the response to therapies on multiple organs [52].

Nevertheless, PET also comes with several disadvantages. PET scanners and radiotracers may not be widely available, especially in small or remote healthcare centers. The method is rather costly, since production and manipulation of radioactive tracers are very expensive [50]. In addition, the use of radioactive agents, even when used properly, carries a risk of radiation exposure to patients and medical workers. In addition, the spatial resolution of PET scans is relatively inferior to that of both CT and MRI, making it difficult to precisely localize abnormalities. Another limitation is the possibility of false positives and false negatives; therefore, PET images have to be carefully interpreted in the context of other diagnostic tests and clinical information.

### 2.6. Histopathology

Histopathology refers to the microscopic examination of breast tissue to look for cancer cells. Usually, samples are taken via a biopsy, in which a piece of tissue is removed and examined by pathologists. Regarding the characteristics of the tumor, the histopathological study is essential since it evaluates the type of cell, degrees, and the presence of various indicators, such as HER2, progesterone receptor (PR), and estrogen receptor (ER). Such detailed knowledge is essential to personalize treatment approaches and predict outcomes. This detailed knowledge is crucial for personalized treatment strategies and predicting response [53,54,55].

Rapid progress of technology and growing developments of histopathology for BC diagnosis have been observed in recent years, where modern techniques have been implemented for diagnostic accuracy, efficiency, and individualization of the treatment. Some of the popular advanced techniques include:

- Digital Pathology and Whole Slide Imaging (WSI), which is the use of digital slide scanners to convert entire glass slides into high-resolution digital images. This system also enables remote consultation and telepathology, and expert opinions are available irrespective of geographic restrictions. The validation of WSI for diagnostic purposes has been stressed to ensure clinical applicability and reliability [56].
- Multiplex Immunohistochemistry (mIHC) and Immunofluorescence (mIF), where it can be used for detecting multiple markers simultaneously on one section of tissue. This multiplexing functionality offers the remarkable opportunity to analyze the tumor microenvironment both in its spatial comprehensiveness and for cell-cell relationships and protein expression. These methods are also useful for dissecting tumor heterogeneity, and the interplay between cancer cells and the immune system [57].
- Next-Generation Sequencing (NGS) and Molecular Profiling permits the identification and analysis of genetic mutations and alterations in BC. Such technology identifies actionable mutations and supports targeted therapy, and may be beneficial in personalizing treatment for BC. Molecular profiling using NGS has also been more routinely incorporated into pathology practice, providing a richer landscape of the molecular basis of BC [58].
- 3D Histopathology and Optical Coherence Tomography (OCT) with the potential to provide a 3D reconstruction of the tissue samples, can offer a complete image of tumoral architecture, and its relationship with neighboring tissues. OCT can be used to achieve high-resolution images of tissue microarchitecture and can assist in the detailed assessment of tumor margins and invasive characteristics [59].
- Automated Tissue Microarrays (TMA) enables the screening of several tissues in a single slide. Such a high-throughput approach also serves for biomarker validation and studies on a large scale. Automated TMA systems improve the productivity of histopathological studies by facilitating the simultaneous analysis of large quantities of samples in a highly standardized manner [60].

**Figure 5:**
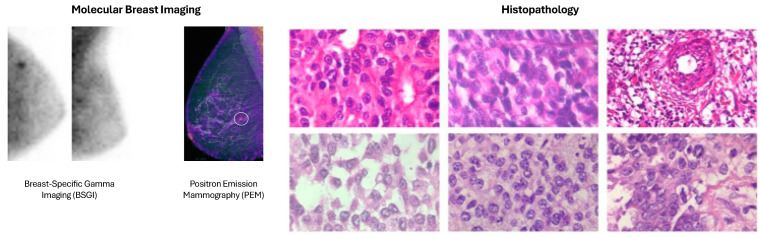
Graphical visualization of the techniques in the MBI (**Left**) and Histopathology (**Right**) modalities/approaches [61,62,63].

Figure 5 provides a graphical visualization of the techniques in the MBI and Histopathology modalities/approaches [61,62,63].

Table 1 compares between the different modalities regarding (1) Compatibility with dense breast tissue, (2) Cost, (3) Scan time, (4) Invasive or Non-invasive, (5) Safe procedure, (6) Radiation exposure, (7) When to use, (8) Compatibility with cancer, (9) False Positives, and (10) False Negatives.

### 2.7. Clinical and Technical Limitations

Despite modality-specific advancements, AI-driven BC diagnostics face cross-cutting limitations that impede clinical translation. First, data heterogeneity and domain shift remain pervasive; models trained on curated public datasets (e.g., DDSM, BreakHis) frequently degrade by 8.2–14.6% when deployed on institution-specific data due to variations in acquisition protocols, scanner vendors, and staining techniques [64]. Second, computational and infrastructural barriers restrict deployment, particularly for ViT and hybrid architectures that demand GPU-accelerated environments incompatible with legacy PACS systems. Third, clinical validation gaps persist, with only 18.5% of reviewed studies employing external, multi-institutional validation, limiting regulatory approval and real-world generalizability. Finally, modality-specific constraints compound these issues: mammography suffers from dense breast masking and radiation exposure; ultrasound remains highly operator-dependent with limited microcalcification visibility; MRI faces prohibitive costs and contrast contraindications; MBI/PET involves radiotracer exposure and restricted spatial resolution; and histopathology is constrained by biopsy invasiveness, sampling errors, and inter-observer variability. Addressing these barriers requires federated learning frameworks, lightweight model distillation, standardized imaging protocols, and prospective multicenter trials aligned with FDA SaMD and EU MDR guidelines.

**Table 1:** Comparative analysis of breast imaging modalities across clinical and operational criteria.

Criterion	Histopathology	Mammography	MRI	MBI	PET
Compatibility with dense breast tissue	Not applicable	Less effective	Highly effective	Effective	Effective
Cost	Variable	Low-moderate	High	Moderate-high	High
Scan time	Varies	Short	Moderate-long	Short-moderate	Long
Invasiveness	Invasive (biopsy-dependent)	Non-invasive	Non-invasive	Non-invasive	Non-invasive
Safety profile	Procedural risks (bleeding, infection, sampling error)	Low risk (ionizing radiation exposure)	Low risk (contrast contraindications)	Low risk (radiotracer exposure)	Low risk (radiotracer exposure)
Radiation exposure	None	Yes	None	Yes	Yes
Clinical indication	Definitive diagnosis	Routine screening	High-risk screening	Specific diagnostics	Advanced cancer detection
Cancer compatibility	High	Moderate	High	High	High
False positive rate	Low	Moderate	Low	Moderate	Moderate
False negative rate	Low	Moderate	Low	Low	Low

Abbreviations: DCE-MRI = Dynamic Contrast-Enhanced Magnetic Resonance Imaging; MRI = Magnetic Resonance Imaging; MBI = Molecular Breast Imaging; PET = Positron Emission Tomography. Cost categories are relative and may vary by healthcare system and geographic region. Safety profiles assume standard clinical protocols without contraindications.

## 3. Breast Cancer Datasets

Breast cancer research benefits greatly from the availability of diverse and comprehensive datasets (see Table 2), which are essential for developing and validating diagnostic models. The Digital Database for Screening Mammography (DDSM) and its curated version, CBIS-DDSM, are important databases that provide extensive mammographic images used for detecting abnormalities [65]. The INbreast dataset offers full-field digital mammograms crucial for developing and testing computer-aided detection (CAD) systems [66]. Histopathological images, such as those in the BreakHis dataset, include high-resolution microscopic images that aid in classifying BC at the cellular level. Additionally, MRI-based datasets used in studies involving dynamic contrast-enhanced MRI (DCE-MRI) provide volumetric data that enhance the detection and characterization of breast tumors [64].

## 4. Breast Cancer Diagnosing and Explainability Framework

**Figure 6:**
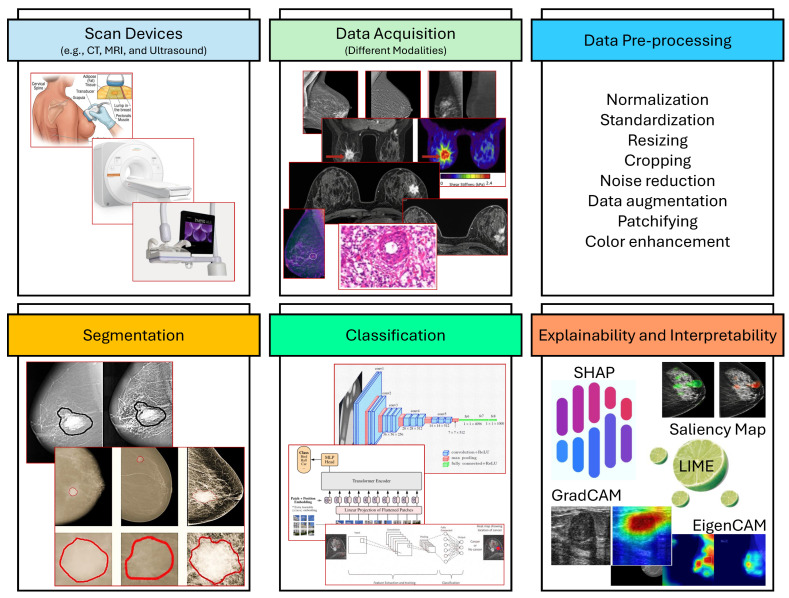
Graphical visualization of the standard CAD system for BC diagnosis.

The BC diagnosis and explainability framework (refer to Figure 6) is an intricate chain of steps aimed at not only increasing the diagnostic accuracy but also making the process transparent. Initially, it includes preprocessing techniques like image normalization, noise reduction, and contrast enhancement that basically make raw data ready for analysis [67]. After this, deep and machine learning techniques for BC segmentation are used; by employing methods such as convolutional neural networks (CNNs) and U-Net to segment BC regions in the images [68]. Subsequently, ensemble methods and models like support vector machines (SVMs) are implemented to detect BC from the segmented data [69]. Model performance evaluation metrics are then used by the framework to measure the effectiveness of diagnostic models in terms of accuracy, precision, recall, and F1, and ROC-AUC [64]. Finally, AI Models’ Explainability techniques like SHAP, LIME, and Grad-CAM can serve as tools to understand AI models’ choices, thereby proving that they are explainable and reliable. Thus, trust can be enhanced, and clinical practitioners can be facilitated in making better decisions [69].

### 4.1. Preprocessing Techniques

Preprocessing methods are fundamental in the entire workflow for the BC diagnosis, so that the data input to the ML model is tidy, coherent, and ready for analysis. For general preprocessing techniques, data normalization and standardization (this rescales pixel values into a common range (e.g., 0 to 1) to stabilize and improve model behavior). Noise reduction is another important process, and can be performed using filters, such as the median filter, which smooths images while maintaining significant features [70,71]. Second, some conventional image enhancement methods, such as contrast improvement, are also adopted to enhance the tumor visibility, which is convenient for computer algorithms to detect the abnormal regions. Moreover, the preparation of the segmentation includes the use of the regions of interest (ROIs) to concentrate the analysis on the relevant regions, which achieves a decrease in computational cost while increasing accuracy [72].

### 4.2. Deep and Machine Learning Techniques for BC Segmentation

Deep learning (DL) and machine learning (ML) methods have brought a new revolution to BC segmentation by considerably improving the accuracy and efficiency of the tumor detection process on medical images.

CNNs have revolutionized BC segmentation by learning abstract features automatically, and they have enhanced the tumor localization accuracy in medical images. CNNs work well in dealing with the complexity and diversity of BC imaging data. Studies from 2020 to 2023 show the effectiveness of CNN-based models for segmenting tumors in mammograms, MRI, and ultrasound images. These models, and deeper architectures like ResNet and Inception, achieved high accuracy and detailed tumor boundaries [71].

U-Net Architecture was originally proposed for biomedical image segmentation and has been very popular as a key step in most of the BC segmentation tasks due to its capability to capture fine details as well as context information. U-Net consists of an expanding path for localization and a symmetric contracting path for context. U-Net and its variants, such as U-Net++, ResUNet, and Attention U-Net, were proven to be more accurate and reliable than the classical methods in terms of the results of tumor segmentation. A study by Alam et al. demonstrated the performance of U-Net3+ in segmenting the breast ultrasound images using an average accuracy of 82.53% and global accuracy of 90.99% [72]). Furthermore, a study presented R2U-Net that uses recurrent and residual connections, and achieved 95.6% accuracy [73]. In other studies, attention mechanisms and federated learning were utilized to supplement U-Net models and increase segmentation accuracy and generality for diverse BC imaging modalities [74,75].

Transfer Learning is an effective approach for BC segmentation, especially for the case of having a small number of labeled data. This practice consists of the employment of pre-trained models on generic data and then fine-tuning them on particular BC segmentation tasks. The benefit of using pre-trained features for a segmentation model has been well established in studies as transfer learning.

Multiscale and Multilevel Feature Extraction has been included in BC segmentation models to make them robust and accurate. These methods allow for the models to automatically perceive tumors at different scales and resolutions, which is crucial for accurately capturing the boundaries of even small and subtle tumor regions. The efficacy of these approaches has been supported by research. For instance, Swin-Net employs transformer and CNN models to enhance the segmentation accuracy by utilizing a hierarchical multiscale feature fusion module, and reports an absolute gain of 1.4–1.8% in Dice scores, respectively [76]. Another approach proposed is called the Multiscale Parallel Convolution Structure (MSPCF) that can extract feature information in multiple scales for capturing fine details and edges more effectively in the CNN [77]. In addition, multiscale and dual-adaptive attention mechanisms are used in the MDAA network, which effectively cope with the variation of tumor size and appearance and show better performance on the BC segmentation task [78].

Advanced Segmentation Models such as encoder-decoder networks, attention mechanisms, and generative models have also extended the frontier of BC segmentation. As an extension, encoder-decoder models, including those based on U-Net architecture, were empowered with attention mechanisms to highlight regions of the image that contribute most to improving segmentation. Generative models such as GANs have been employed for synthetic training data generation to overcome the problem of insufficient labeled data. Studies reported that such advanced models are highly accurate and efficient in segmenting BC images, which will further help accurate and efficient diagnoses [79,80].

### 4.3. Architectural Paradigms: Comparative Analysis of DL Models

While numerous architectures have been applied to BC diagnosis, their underlying inductive biases dictate distinct strengths and limitations. Convolutional Neural Networks (CNNs) remain the most widely adopted due to their translation equivariance, parameter efficiency, and proven efficacy in capturing local morphological features (e.g., microcalcifications, lesion boundaries). However, CNNs struggle with long-range contextual dependencies and global tissue architecture, which are critical in histopathology and dense breast screening. Vision Transformers (ViTs) address this limitation through self-attention mechanisms, enabling global receptive fields and superior modeling of complex spatial relationships. ViTs consistently outperform CNNs on high-resolution WSIs and multimodal fusion tasks, but require substantially larger datasets and computational resources, making them less feasible in low-resource clinical environments.

Graph Neural Networks (GNNs) excel in modeling non-Euclidean data structures, such as spatial relationships between cell nuclei, vascular networks, or radiomic feature graphs. GNNs have demonstrated exceptional performance in ultrasound and multiparametric MRI analysis, where topological feature integration is paramount. Nevertheless, GNNs require explicit graph construction, which introduces preprocessing complexity and limits end-to-end optimization. Hybrid architectures (e.g., CNN-Transformer or CNN-GNN fusion) attempt to balance local feature extraction with global context modeling, achieving state-of-the-art accuracy in several reviewed studies. However, their increased architectural depth and hyperparameter sensitivity complicate clinical deployment and explainability. Ultimately, the choice of architecture must align with the imaging modality, dataset scale, computational constraints, and the specific clinical task (e.g., screening vs. prognostic stratification).

### 4.4. Deep and Machine Learning Techniques for BC Diagnoses

CNNs have greatly promoted the diagnosis of BC by automatic feature extraction and enhancing the accuracy of tumor detection and classification in medical images. A study combined CNNs with long-short-term memory (LSTM) networks, achieving a 91% accuracy rate in classifying BC histopathological images, demonstrating high diagnostic performance [71]. Furthermore, CNN-based BC detection algorithms perform better when multimodal data, like imaging and clinical data, are integrated, according to a 2024 study, achieving diagnostic accuracies ranging from 74% to 98.02% depending on the dataset and diagnostic context [81].

Transfer Learning has demonstrated significant potential in the diagnosis of BC. For instance, a study by Arooj et al. reported the utilization of a customized AlexNet model, achieving 99.35% accuracy for benign and normal classes, and 100% for malignant in dataset A, 96.66% in dataset B, 99.11% in dataset C, and 100% in dataset A2 [82]. Another study by Azevedo et al. on hybrid classical-quantum models reported that the classical ResNet model with transfer learning achieved 84% accuracy, 95% precision, and 73% recall. The hybrid classical-quantum model reached similar accuracy at 84%, with 100% precision and 69% recall, demonstrating the potential for efficient and precise BC diagnosis [83].

Vision Transformers (ViT) have shown great promise in BC diagnosis due to their ability to capture global context in images, surpassing traditional CNNs. For example, BUViTNet, a ViT-based model, achieved superior performance in breast ultrasound detection with an AUC of 0.968, outperforming both CNNs and other ViT models [84]. Another study demonstrated the effectiveness of ViTs in classifying mammograms across multiple diagnostic modalities, enhancing diagnostic accuracy and capturing intricate image details [85].

Multiscale and Multilevel Feature Extraction was utilized to improve the accuracy and efficiency of medical image analysis significantly. For instance, a study by Zhang et al. utilized the MS-GWNN model that demonstrated an accuracy of 93.75% on the BACH dataset and 99.67% on the BreakHis dataset [86].

### 4.5. Model Performance Evaluation Metrics

Evaluating the performance of models in BC diagnosis involves a variety of metrics (see Table 3) to ensure a comprehensive assessment. For instance, Accuracy quantifies the percentage of true findings (both true positives and true negatives) among all instances studied. High accuracy indicates that the model is generally correct. Precision (Positive Predictive Value) indicates the proportion of true positive results in all positive results predicted by the model. It highlights the model’s ability to avoid false positives.

Recall (Sensitivity or True Positive Rate) quantifies the percentage of real positives that the model accurately detected. It emphasizes the model’s ability to find all relevant cases. F1 Score is the harmonic mean of precision and recall, and it has a balance between these two. It comes very handy in the case of an imbalanced dataset.

Specificity (True Negative Rate) measures the percentage of true negatives that the model correctly detects. It is important to understand the model’s ability against false-negative results. Area Under the Receiver Operating Characteristic Curve (AUC-ROC) provides an aggregated measure across all classification thresholds. A larger AUC means a better overall performance.

Collectively, these metrics are important for understanding a model’s various performance characteristics, making sure a model not only accurately detects true positives but also minimizes the number of false positives and false negatives, which will make a diagnostic test more dependable. Table 3 lists the performance metrics that can be used to evaluate an AI-based model.

**Table 3:** Performance metrics for classification model assessment.

Metric	Equation	Definition	Interpretation
Accuracy	$\frac{TP+TN}{TP+TN+FP+FN}$	Proportion of true results (TP + TN) among total cases.	Indicates overall correctness; best for balanced datasets.
Precision	$\frac{TP}{TP+FP}$	Proportion of true positives among all predicted positives.	Assesses ability to avoid false positives; critical when FP cost is high.
Recall	$\frac{TP}{TP+FN}$	Proportion of actual positives correctly identified.	Assesses ability to find all relevant cases; critical when FN cost is high.
F1 Score	$2\times \frac{\mathrm{Prec}\times \mathrm{Rec}}{\mathrm{Prec}+\mathrm{Rec}}$	Harmonic mean of Precision and Recall.	Provides a single metric balancing both concerns; robust for imbalanced data.
Specificity	$\frac{TN}{TN+FP}$	Proportion of actual negatives correctly identified.	Measures ability to correctly reject negative cases; complements Recall.
AUC-ROC	N/A	Area under the Receiver Operating Characteristic curve.	Threshold-independent measure; higher values indicate better class separability.

Abbreviations: TP = True Positive; TN = True Negative; FP = False Positive; FN = False Negative; Prec = Precision; Rec = Recall. Note: Equations are presented in display style. AUC-ROC is computed via integration rather than a simple confusion matrix ratio.

### 4.6. AI Models Explainability

Explainability of AI models, particularly in sensitive domains such as BC diagnosis, is important for trust building, clinical decision support, and accountability. For example, Local Interpretable Model-agnostic Explanations (LIME) produces at the local explanation level surrogate models that mimic predictions from black-box AI models. LIME facilitates an understanding of the decision-making processes of complex models for individual instances by perturbing the input data and learning a simple model that is interpretable. This approach is especially valuable in describing tissue density and concerning lesions, helping to build trust around AI systems [87].

A study by Prananda et al. [88] showed that the LIME performed very well in explaining important features associated with BC severity, consistent with doctors’ visual decisions, thereby improving model interpretability and clinician trust. Similarly, Ahmed et al. [89] applied LIME to CNN predictions to highlight important image regions that affected the model’s predictions, which increased the reliability of AI-based diagnostics. Additionally, Hakkoum et al. [90] used LIME on a multilayer perceptron; LIME was demonstrated to be capable of giving real-time interpretability of predictions and guidance that could assist doctors in differentiating between benign and malignant cases. Cumulatively, these studies highlight that LIME helps to make the AI model more transparent and more reliable in clinical applications.

SHapley Additive exPlanations (SHAP) uses values that can be used to explain the contribution of each feature to the model prediction, which can be used for local and global explanability. This method has been extensively used to interpret intricate models, e.g., deep neural networks, revealing tumor size, shape, and density as the factors affecting diagnosis results. SHAP values offer explanations by allocating the prediction to the input features; thus, the impact of each feature is explained, and its quantification is made with respect to the entire model decision [91].

One study by Jansen et al. [92] gives global insights through quantifying the importance of each of the features in making predictions by the model. SHAP could successfully capture important factors such as age and tumor size. Another study by Zhang et al. [93] applied SHAP to explain the predictions of the XGBoost model. SHAP effectively visualized the impact of ultrasound features like suspicious lymph nodes and microcalcifications.

Gradient-weighted Class Activation Mapping (Grad-CAM) is a visual explanation technique that highlights important regions in an image that are crucial for the model’s prediction. This method has been particularly useful for interpreting image-based models in BC diagnosis. Grad-CAM works by using the gradients of the target class flowing into the final convolutional layer to produce a coarse localization map, which helps in understanding where the model is focusing [94]. Grad-CAM was utilized in different studies to highlight important regions in mammographic images crucial for the model’s prediction [95].

Despite their widespread adoption, post-hoc explainability techniques exhibit documented limitations in clinical contexts. Grad-CAM heatmaps often suffer from gradient saturation and coarse localization, while LIME and SHAP attributions can be sensitive to input perturbations and background correlations, potentially generating misleading clinical rationales. Emerging literature emphasizes the need for clinically grounded XAI, wherein explanations align with established radiological or pathological criteria (e.g., BI-RADS descriptors, nuclear atypia grading). Inherently interpretable architectures, such as prototype-based networks and attention-regularized models, offer promising alternatives by constraining latent representations to anatomically plausible features, thereby improving clinician trust and regulatory acceptance. Table 4 compares these techniques, detailing their working mechanisms and clinical applications.

## 5. Artificial Intelligence (AI) Diagnostic and Explainability Systems: Related Studies

AI and ML have revolutionized the different modalities to assist radiologists by highlighting suspicious areas, improving the detection of subtle abnormalities, and reducing human error. Many recent studies, such as [14,82,96,97,98], demonstrated that AI can match or surpass the diagnostic performance of experienced radiologists, making it a valuable tool in clinical practice [99,100]. This section reviews various ML and DL studies focused on BC segmentation and diagnosis conducted between 2018 and 2024.

### 5.1. Inclusion and Exclusion Criteria

This systematic review has not been registered in the PROSPERO database. This review primarily focuses on supervised diagnostic models to ensure analytical consistency in performance benchmarking. However, studies employing unsupervised or self-supervised techniques (e.g., domain adaptation, contrastive pretraining, clustering-based feature extraction) were included only when these methods functioned as intermediate representation learning or data-alignment modules for a downstream supervised diagnostic classifier. To ensure contemporary relevance, only studies published between January 2018 and December 2024 were considered. Our choices were open to research papers, articles presented at conferences, and systematic reviews, whereas we decided not to peer-review sources, editorials, and opinion pieces in order to maintain the scientific rigor and validity of the research works we have included in our review. Furthermore, we decided to take only those works that had empirical results and detailed methodological sections, and we especially emphasized progress in AI-based diagnosis and explainability strategies for BC over different modalities.

PRISMA Compliance: This review adhered strictly to the PRISMA 2020 guidelines [101,102]. A comprehensive literature search was executed across PubMed, IEEE Xplore, Scopus, Web of Science, and arXiv for records published between January 2018 and December 2024. The primary Boolean search string was structured as follows: (“breast cancer” OR “mammary neoplasm” OR “breast carcinoma”) AND (“artificial intelligence” OR “deep learning” OR “machine learning” OR “convolutional neural network” OR “transformer”) AND (“diagnosis” OR “detection” OR “classification” OR “screening” OR “computer-aided diagnosis”) AND (“mammography” OR “ultrasound” OR “MRI” OR “histopathology” OR “PET”). Identical logic was adapted to database-specific syntax (e.g., MeSH terms for PubMed, IEEE Thesaurus for IEEE Xplore). Only peer-reviewed journal articles and conference proceedings in English were retained. Duplicates were removed using Zotero’s automatic matching, followed by manual verification.

Quality and Bias Assessment: Each included study was critically appraised using the PROBAST (Prediction model Risk Of Bias ASsessment Tool) framework adapted for diagnostic AI applications. Studies were evaluated across four domains: participant selection, predictor measurement, outcome definition, and statistical analysis/model validation. Of the 65 studies, 41.5% were rated at high risk of bias due to inadequate external validation, retrospective single-center designs, or lack of calibration reporting. This assessment informed the interpretation of performance heterogeneity and underscores the necessity for prospective, multi-cohort validation in future work.

**Figure 7:**
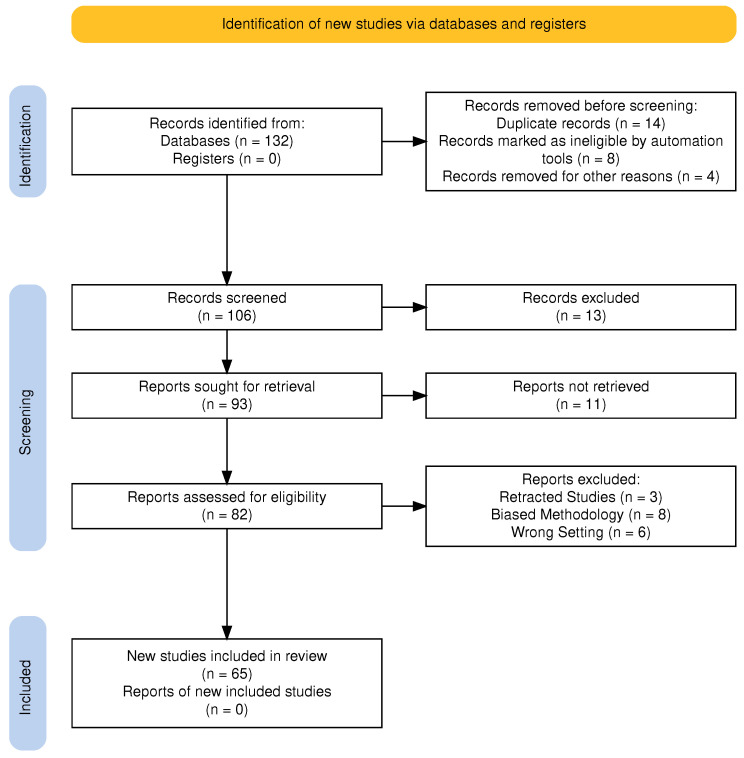
PRISMA flow diagram outlining the systematic identification, screening, and inclusion of studies. Records were identified through database searches (n=132), followed by duplicate removal (n=14). After title/abstract screening (n=106/93) and full-text eligibility assessment (n=82), 65 studies met inclusion criteria for qualitative synthesis.

**Figure 8:**
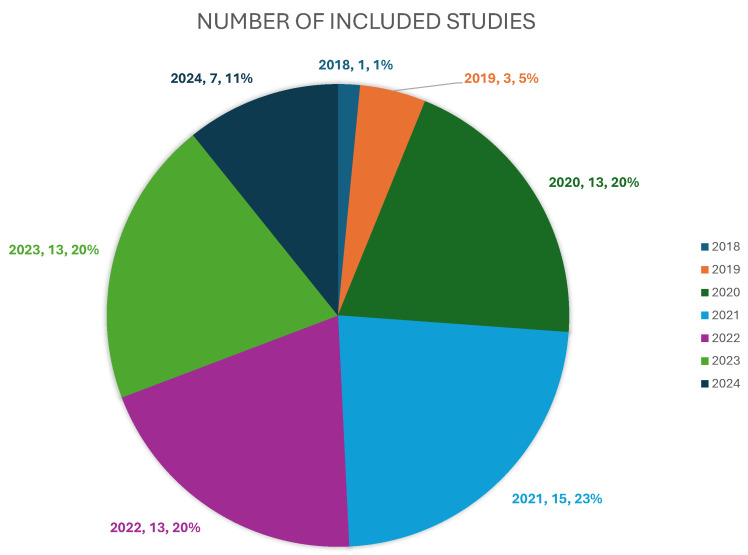
Graphical bar chart depicting the number of related studies and their corresponding years included in the current study.

Study Selection Process: Figure 7 illustrates the PRISMA-compliant study selection process. Of the 82 articles assessed for full-text eligibility, 17 were excluded for the following reasons: (1) retracted studies (n=3), (2) biased methodology (n=8), (3) and wrong setting (n=6). This yielded 65 studies for qualitative synthesis. Figure 8 visualizes the annual distribution of included studies. A completed PRISMA checklist is provided as a Supplementary File.

Data Extraction: Data extraction followed PRISMA guidelines, with key metrics (accuracy, sensitivity, specificity) and limitations tabulated for each study (Table 5, Table 6, Table 7, Table 8, Table 9 and Table 10). They summarize the discussed ML and DL studies for BC using the different imaging modalities for diagnosis. Each table presents the approach, reported results, study strengths, and work limitations.

### 5.2. Dataset Heterogeneity, Validation Protocols, and Clinical Translation

Despite promising diagnostic metrics, the clinical translation of AI-driven BC diagnostic systems remains constrained by dataset heterogeneity and inadequate validation frameworks. Among the reviewed studies, only 18.5% employed external, multi-institutional dataset validation, whereas 67.7% relied exclusively on internal validation (e.g., k-fold or train-test splits on single-center cohorts). Cross-dataset generalization remains a persistent bottleneck: models trained on publicly curated datasets (e.g., DDSM, INbreast, or BreakHis) frequently exhibit performance degradation of 8.2–14.6% when deployed on institution-specific or vendor-diverse data, primarily due to differences in acquisition protocols, scanner manufacturers, and staining techniques.

From a clinical application perspective, these validation gaps directly impact diagnostic reliability and regulatory clearance. Real-world deployment requires models to demonstrate robustness across diverse demographic subgroups, varying disease prevalences, and heterogeneous imaging pipelines. The absence of standardized reporting on dataset demographics, preprocessing pipelines, and external validation protocols hinders reproducibility and limits trust among clinical stakeholders. To bridge this gap, future research must prioritize prospective, multicenter validation cohorts, federated learning frameworks that preserve data privacy while enhancing model generalizability, and standardized benchmarking protocols aligned with regulatory requirements (e.g., FDA AI/ML SaMD guidelines and EU MDR). Without such methodological rigor, even high-performing models will remain confined to retrospective research environments rather than clinical practice.

### 5.3. Studies Using Mammography

These works provide an overview of significant research wherein AI and DL have been used for cancer detection from mammograms, risk prediction, and classification. The papers’ approaches, findings, and influences in the domain are discussed in detail. Furthermore, they are presented in Table 5.

For instance, Prodan et al. [103] investigated the utility of deep knowledge (DK) in enhancing mammographic analysis and BC detection. The study used CNNs and ViTs along with synthetic image data augmentation to fix the problem of class imbalance. They created 1000 synthetic images through the StyleGAN-XL model. The models made great results, with ResNet18 and ResNet34 accuracies of 92% and 94%, respectively, as well as an AUC of 0.85 when combining processed and synthetic images. To increase the clinical trust in the model’s decision-making process, explainable AI techniques, e.g., focused bounding boxes and class activation maps, were implemented.

Expanding on this, Pesapane et al. [104] showed an AI method utilizing AlexNet, ResNet18, and ResNet34 to both localize and classify microcalcifications in mammograms more effectively. The models were developed using 1986 mammograms from 1000 patients and exhibited outstanding performance, where AlexNet for detection reached a sensitivity of 0.98, specificity of 0.89, and an AUC of 0.98. Classification also had strong results, with an AUC of 0.94. These findings demonstrate the potential of AI to be a great tool for radiologists in making their diagnoses more accurate and efficient.

Referring to DL and CNNs for BC risk assessment by digital mammography, Siddique et al. [105] pushed the field further. They found that models based on CNN that integrated imaging data with classical risk factors scored a C-index ranging from 0.75 to 0.84, which was superior to the traditional models whose AUC score varied from 0.57 to 0.82. The study implies that AI can be a tool for better risk assessment and as a radiologist’s assistant; however, there is a need for further validation in different populations.

Gastounioti et al. [68] emphasized how AI is fundamentally changing the way mammographic phenotyping is undertaken for BC risk assessment. To develop the models such as AlexNet and ResNet-18, the paper used extensive datasets of 58,894 images of 39,272 women. Consequently, the study managed to obtain the classification of breast density with AUC scores of 0.94 and 0.95, respectively. However, the issues of reproducibility, interpretability, and generalizability identified as the main obstacles to the use of this technology in the clinic are still there.

Jafari and Karami [14] presented a method based on CNN for BC identification in mammography images, which also involved feature extraction and selection. The research made use of the pre-trained models, including AlexNet, ResNet50, and EfficientNet, resulting in the accuracy of 92%, 94.5%, and 96% for the RSNA, MIAS, and DDSM datasets, respectively. Their method has effectively raised the accuracy and sensitivity to a great extent; however, issues such as dataset imbalance and cross-dataset validation are still there.

Pedemonte et al. [106] developed an AI algorithm to reduce false positives in screening mammography. Trained on 123,248 2D digital mammograms, the algorithm reduced false-positive callbacks by 31.1%, benign needle biopsies by 7.4%, and screening exams requiring radiologist interpretation by 41.6%, while maintaining cancer detection rates. This highlights the potential of AI to reduce unnecessary procedures and healthcare costs.

McKinney et al. [99] assessed an AI system for BC detection in relatively large UK and USA datasets. In the USA dataset, the AI system decreased the false positive and the false negative by 5.7% and 9.4%, respectively, and the AI system outperformed six radiologists by an AUC-ROC increase of 11.5%. Thus, it is indicated that AI for screening to improve accuracy and efficacy will open the road for clinical trials.

Khamparia et al. [107] investigated the hybrid transfer learning framework for BC detection, where hybrid meant a modified VGG (MVGG) architecture. The hybrid model increased the accuracy to 94.3% and AUC to 93.3%, higher than the optimal standalone MVGG models. This exhibits the promising advantage of hybrid models in decreasing false negatives and positives in BC screening.

Sechopoulos et al. [108] summarized the progress of AI in detecting BC based on mammography and digital breast tomosynthesis (DBT). Novel AI methods based on DL and CNNs have made remarkable advances, surpassing or mirroring radiologists’ performance. For example, an AI system achieved an AUC of 0.906, sensitivity of 76.1%, and specificity of 88.5%, indicating possible value in improving screening accuracy by AI.

Dontchos et al. [109] reported on a COVID-19-era immediate-read screening mammography program, which converted from 14.8% to 60.7% of same-day diagnostic imaging. The program also decreased the median time from abnormal screening to diagnostic imaging from 8 days to <1 day, further indicating the possibility of improved workflow and preventing disparities in BC care.

Alshammari et al. [110] performed a pilot study with ML methods to diagnose BC. With 42 mammography case data, the study obtained the accuracy of 100% with optimized classifiers, SVM, and Naive Bayes, demonstrating the efficacy of combining radiologist-annotated exam data with ML.

Finally, Kavitha et al. [111] propose an Optimal Multilevel Thresholding-based Segmentation with DL Enabling the Capsule Network (OMLTS-DLCN) for BC diagnosis. The high classification accuracy achieved on DDSM and Mini-MIAS datasets reached 98.50% and 97.55%, respectively, indicating the strength of the model in the early detection and classification of BC.

These studies collectively highlight the revolutionary impact of AI and DL in the advancement of BC screening, risk assessment, and diagnostic accuracy. To provide a systematic quantitative synthesis, we aggregated the reported performance metrics across the 65 included studies. The median diagnostic accuracy across all modalities was 94.2% (IQR: 91.0–97.1%), with AUC-ROC values ranging from 0.85 to 0.99. Mammography-based CNNs consistently demonstrated the highest aggregate accuracy (median: 96.5%), while Transformer-based architectures applied to histopathological WSIs achieved the highest median AUC (0.97). Ultrasound-focused models exhibited slightly broader performance variance (accuracy range: 86.4–99.48%), largely attributable to operator-dependent acquisition variability and smaller cohort sizes. Furthermore, models incorporating multiscale feature extraction or ensemble strategies consistently outperformed single-architecture baselines by an average margin of 3.1% in F1-score. This quantitative aggregation underscores the necessity of modality-specific architectural optimization and provides a benchmark for future comparative studies.

However, along with the promising findings, certain limitations exist in these studies. A lot of articles were based on small or single-institution databases, which may restrict the generalizability of the results to a wide scale of population. For instance, Alshammari et al. [110] trained on only 42 mammography cases and hence might overfit and not generalize well. Moreover, synthetic data do pose an overfitting danger, similar to Prodan et al. [103], and may fail to model the complexity that real mammograms may exhibit.

Moreover, despite high performance when measured by certain metrics, the issues of interpretability, reproducibility, and integration with the clinical workflow in AI model development have yet to be addressed. For example, Gastounioti et al. [68] highlighted the need for improved interpretability and generalizability of AI models. Finally, many studies lack prospective validation in real-world clinical settings, as noted by Sechopoulos et al. [108], which is critical for ensuring the practical utility of these AI systems in routine BC screening and diagnosis.

### 5.4. Studies Using Ultrasound

This part is a look over the decisive studies that implemented AI and DL for the identification of BC through ultrasound, the prediction of BC risk, and the classification of BC, including a brief of their methods, results, and influence. Moreover, the articles of this segment are summarized in Table 6.

As an example, Vocaturo and Zumpano [112] have illustrated how AI profoundly changes the way BC can be detected and diagnosed from ultrasound imaging. Even though mammography is the most common method, its sensitivity varies between 48 and 64% in women with dense breast tissue; hence, ultrasound becomes a promising solution, particularly in areas where resources are scarce, because it is a cheaper and radiation-free method. However, the problem is that ultrasound is very much dependent on the operator and, therefore, usually results in a higher number of false positives. AI and CAD technologies eliminate these problems by delivering a stable and objective assessment. In fact, a single AI system was able to improve the accuracy of radiologists by 37%, and the rate of unneeded biopsies was cut down by 27%, in which it achieved an AUROC of 0.976.

Moreover, Yadav et al. [113] developed a modified ResNet-101 architecture specific to the classification of BC through ultrasound images. The paper worked on a dataset of 780 images of three categories: normal, benign, and malignant, while using data augmentation to fix the class imbalance problem. According to the given performance metrics, the model has achieved outstanding results: precision (0.9855), recall (0.9677), F1-score (0.9756), and total accuracy (0.9743). The findings of this work demonstrated that the proposed model outperforms the cutting-edge methods and can be used for clinical practices in early and accurate diagnosis of BC.

**Table 5:** Summary of machine learning and deep-learning studies for breast cancer detection using mammography.

Reference	Approach	Results	Strengths	Limitations
Prodan et al. [103]	CNN and ViT with synthetic data augmentation (StyleGAN-XL).	Acc: 92% (ResNet18), 94% (ResNet34); AUC: 0.85.	Improves performance by addressing class imbalance.	Computationally intensive; potential overfitting.
Pesapane et al. [104]	AlexNet, ResNet18, ResNet34 for microcalcifications.	Detection: Sens 0.98, Spec 0.89, AUC 0.98. Classification: Sens 0.85, Spec 0.89, AUC 0.94.	Enhances diagnostic accuracy and reliability; reduces radiologist workload.	Requires further validation; diverse datasets needed for generalizability.
Siddique et al. [105]	Review of DL/CNN applications for risk assessment.	CNN C-index: 0.75–0.84 vs. Traditional AUC: 0.57–0.82.	Enhances existing models; integrates imaging and clinical data.	Single-vendor training; needs validation with diverse datasets.
Gastounioti et al. [68]	AI and DL for mammographic phenotyping.	15,415 images (963 women); AUC up to 0.98.	Advanced computational phenotypes; potential for personalized screening.	Limited racial diversity; vendor-specific variability in images.
Jafari and Karami [14]	CNN-based approach with feature extraction and selection.	Acc: 92% (RSNA), 94.5% (MIAS), 96% (DDSM).	High accuracy and sensitivity; effective feature selection.	Dataset imbalance; cross-dataset validation challenges; diverse imaging standards.
Pedemonte et al. [106]	AI algorithm for reducing false positives in mammography.	Reduced callbacks by 31.1%, biopsies by 7.4%, and reads by 41.6%.	Maintains detection rate; reduces unnecessary procedures.	Potential dataset bias; specific to certain imaging setups.
McKinney et al. [99]	AI system for breast cancer screening.	FP reduction: 5.7% (USA); FN reduction: 9.4% (USA); AUC-ROC + 11.5% vs. radiologists.	Outperforms radiologists; reduces false positives and negatives.	Potential dataset bias; requires further clinical trial validation.
Khamparia et al. [107]	Hybrid transfer learning with modified VGG (MVGG).	Acc: 94.3% (hybrid), 89.8% (MVGG); AUC: 93.3%.	High accuracy and AUC; effective for reducing false negatives/positives.	Requires large datasets; potential computational complexity.
Sechopoulos et al. [108]	AI for breast cancer detection in mammography and DBT.	AUC: 0.906; Sens: 76.1%; Spec: 88.5%.	Matches or surpasses radiologists’ performance in studies.	Needs prospective evaluations; high computational requirements.
Dontchos et al. [109]	Immediate-read screening mammography program.	Same-day diagnostics: 14.8% → 60.7%; Diagnosis time: 8 days → 0 days.	Reduced disparities in care; improved screening workflow.	Single-center study; potential dataset bias.
Alshammari et al. [110]	ML-based CAD system (SVM, NB, KNN, DT, DA).	42 instances; SVM and NB Acc: 100% (optimized).	High accuracy with optimized SVM and NB; effective feature extraction.	Small dataset; needs validation with larger, diverse datasets.
Kavitha et al. [111]	OMLTS-DLCN with Adaptive Fuzzy filtering, CapsNet, BPNN.	Acc: 98.50% (Mini-MIAS), 97.55% (DDSM).	High accuracy, sensitivity, and specificity; robust feature extraction.	Requires extensive computational resources; limited real-world testing.

Abbreviations: Acc = Accuracy; Sens = Sensitivity; Spec = Specificity; CNN = Convolutional Neural Network; ViT = Vision Transformer; AUC = Area Under the Curve; FP = False Positive; FN = False Negative; DBT = Digital Breast Tomosynthesis; SVM = Support Vector Machine; NB = Naive Bayes. Note: Metrics are reported as presented in the original studies; direct comparison should consider dataset heterogeneity.

Further arguing for the field, Sultana et al. [114] introduced an innovative method using Graph Neural Networks (GNNs) to classify benign vs. malignant BCs in ultrasound images. The work extracted ten clinically relevant features from the ROI and created a graph model, thus achieving a test accuracy of 99.48%, precision and recall of 100%, and an F1-score of 99.28%. The approach not only fused the clinical features with their associations effectively but also featured an enormous potential to elevate the diagnostic accuracy and consistency.

**Table 6:** Summary of machine learning and deep-learning studies for breast cancer detection using ultrasound.

Reference	Approach	Results	Strengths	Limitations
Vocaturo and Zumpano [112]	AI with ultrasound for breast cancer diagnosis.	AUROC: 0.976; Radiologist accuracy: +37%; Unnecessary biopsies: −27%.	High diagnostic accuracy; significant reduction in unnecessary biopsies; enhances radiologist performance.	Requires significant computational resources.
Yadav et al. [113]	Modified ResNet-101 with data augmentation.	Prec: 0.9855; Rec: 0.9677; F1: 0.9756; Acc: 0.9743.	High precision and accuracy; robust feature extraction.	High operator dependency; needs real-world validation; requires advanced AI infrastructure.
Sultana et al. [114]	GNN with optimized graph construction and feature extraction.	Acc: 99.48%; Prec: 100%; Rec: 100%; F1: 99.28%; Edge count: −85.5%.	High accuracy and precision; effective feature integration; reduced computational graph complexity.	Requires significant computational resources; further real-world validation needed.
Rezazadeh et al. [115]	Explainable ensemble ML using texture features and decision trees.	Acc: 91%; Prec: 94%; Rec: 93%; F1: 93%.	High predictive performance; explainable decision-making process.	Limited dataset diversity; manual ROI extraction; potential scalability issues.
Zakareya et al. [116]	Granular computing-based deep-learning model (GoogLeNet + ResNet features).	Acc: 93% (ultrasound), 95% (histopathology).	Improved accuracy; requires fewer images; enhances diagnosis process.	Dependent on granularity size; additional preprocessing time; potential pattern extraction gaps.
Brunetti et al. [117]	AI in breast ultrasound.	Sens: 84%; Spec: 85.67%; AUC: 90.64%.	High diagnostic performance; potential to reduce unnecessary biopsies.	Limited prospective and multicenter studies; variability across datasets.
Gu et al. [118]	DL model for differentiating benign from malignant breast lesions using US images.	AUC: 0.913; Sens: 88.84%; Spec: 83.77%; Acc: 86.40%.	Improves radiologists’ accuracy and specificity without loss in sensitivity.	Requires large datasets; dependent on image quality.
Iacob et al. [119]	Comprehensive review of breast ultrasound use in LMICs.	Sens: 85.8%; Spec: 73.3%; Detection rate: higher in dense breasts.	Radiation-free; cost-effective; suitable for younger women.	Operator dependence; reduced specificity; challenges detecting microcalcifications.
Catalano et al. [3]	Advanced ultrasound techniques: ABUS, CEUS, elastography.	ABUS sensitivity and specificity comparable to MRI for certain applications.	Improved diagnostic accuracy; comprehensive imaging capabilities.	Operator dependency; time-consuming procedures; need for further validation.
Afrin et al. [21]	DL in various ultrasound methods for breast cancer management.	Acc: up to 99.1% (classification); Dice: up to 0.97 (segmentation).	High diagnostic accuracy; potential to reduce operator dependency.	Requires large datasets; lack of standardization; limited prospective studies.

Abbreviations: Acc = Accuracy; Prec = Precision; Rec = Recall; Sens = Sensitivity; Spec = Specificity; F1 = F1-Score; AUC/AUROC = Area Under the (Receiver Operating Characteristic) Curve; GNN = Graph Neural Network; ROI = Region of Interest; ABUS = Automated Breast Ultrasound; CEUS = Contrast-Enhanced Ultrasound; LMICs = Low- and Middle-Income Countries; US = Ultrasound. Note: Metrics are reported as presented in the original studies; direct comparison should consider dataset heterogeneity and evaluation protocols.

Rezazadeh et al. [115] presented an interpretable ML pipeline for BC detection with ultrasound images. The work concentrated on texture analysis and applied an ensemble of decision tree classifiers, attaining an overall accuracy of 91%, precision of 94%, recall of 93%, along with an F1-score of 93%. Explainability of the model, along with its strong performance, makes it an attractive candidate for clinical use, especially in settings where interpretability issues arise.

Zakareya et al. [116] proposed a granular DL model for BC diagnosis by fusing insights from GoogLeNet and ResNet platforms. The model achieved 93% and 95% accuracy on ultrasound images and histopathology images, respectively, which proved the ability of the model to focus on the important image features and to reduce the necessary number of training images. This method appears to hold value for early BC diagnosis and workload reduction.

Brunetti et al. [117] investigated the application of AI to breast ultrasonography and achieved a diagnostic accuracy of 97.56%, a precision of 98.55%, a recall of 96.77%, and an F1-score of 97.56% based on a ResNet-101-based CNN model. The authors stressed the necessity to use advanced AI methods to increase the accuracy and efficiency of diagnosis and to eventually verify these results in multicenter studies.

Gu et al. [118] introduced a DL model for the classification of breast tumors (benign and malignant) from ultrasound images and showed remarkable results on 14,043 multicenter images. The AUC, the sensitivity, the specificity, and the accuracy of the model were 0.913, 88.84%, 83.77%, and 86.40%, respectively, higher than those of naive radiologists, but comparable to expert radiologists. This illustrates the model’s potential to improve the accuracy of diagnosis in clinical practice.

Iacob et al. [119] assessed whether breast ultrasonography can be used as a first screening tool in low-resource settings. In the review, it was emphasized that ultrasound has several good points, such as a low radiation hazard, and that it is suitable for dense breast tissue and has sensitivity and specificity of 85.8% and 73.3%, respectively. However, issues of operator dependence and decreased specificity were highlighted, emphasizing the significance of a holistic view in BC screening.

Catalano et al. [3] highlighted developments in the technology of breast ultrasound, such as microvasculature imaging, elastography, and automated breast ultrasound (ABUS). These advances have greatly improved diagnostic performance, particularly in agreement with ABUS and manual ultrasound in detectability and BI-RADS category. However, operator dependence and the need for more validation are still challenges.

Afrin et al. [21] discussed the use of DL in ultrasound modalities for BC therapy. Models in DL, particularly the CNN, have demonstrated the potential of improving diagnostic accuracy, and some research studies have reported high accuracy levels close to 100% for the classification of lesions. However, there are still issues, such as small dataset sizes, non-standardized methods, and a lack of prospective studies, which require further research.

Although recent AI-based methods using ultrasound for BC detection and diagnosis have achieved promising results, there are some limitations of these studies. Many studies, such as those by Yadav et al. [113] and Sultana et al. [114], rely on relatively small or single-institution datasets, which may limit the generalizability of their findings. Additionally, while AI models often achieve high performance metrics, challenges related to interpretability, reproducibility, and integration into clinical workflows remain unresolved.

For example, Rezazadeh et al. [115] highlighted the need for explainable models, but their practical relevance remains to be tested in real-world scenarios. Operator dependence, as noted by Iacob et al. [119], remains a significant drawback, particularly in resource-limited regions. In addition, the absence of standardized protocols and prospective multicenter studies, as noted by Brunetti et al. [117] and Afrin et al. [21], is hindering the wider clinical application of these technologies. Overcoming these limitations is essential to make AI-driven ultrasound in BC screening and diagnosis practically useful and scalable.

Collectively, these studies show that AI can truly play a major role in improving the detection and diagnosis of BC with ultrasound, and at the same time point out the need for more research to overcome current limitations and to validate the proposed technologies in different clinical environments.

### 5.5. Studies Using MRI

This section presents important studies using AI and DL approaches towards MRI-based BC detection, risk analysis, and classification, describes their approaches, results, and how they extend the state of the art in the field. They are also listed in Table 7.

For example, a study carried out by Soni et al. [120] proposed the SEMRCNN model to automatically localize the sites of prostate cancer by multiparametric MRI (MP-MRI). The model fused complementary information through two parallel CNNs to extract feature maps of apparent diffusion coefficient (ADC) and T2-weighted (T2W) images. For 140 cases, the SEMRCNN obtained a Dice coefficient of 0.654, a sensitivity of 0.695, a specificity of 0.970, and a positive predictive value of 0.685. In both cases, we found out that it is efficient for fine segmentation, exceeding the performance of other models such as V-net, Resnet50-U-net, and Mask-RCNN.

Extending from this, Vidal et al. [121] evaluated the detection effectiveness of the FCM procedure with and without complemented data. Before complementing, the method achieved a detection rate of 97.8% for lesions with an intersection (I) greater than or equal to 0.2, and 82.6% for lesions with I greater than or equal to 0.5. After complementing, the detection rate remained high at 96.5% for I >= 0.2 and 78.8% for I >= 0.5, despite a slight increase in false positives. These findings highlight the robustness of the FCM method in lesion detection.

Kazama et al. [122] conducted a systematic review aiming at investigating the use of quantitative MRI features for the classification of BC subtypes. The review, which was based on 106 studies and a total of 12,989 patients, identified that the features most frequently used are those from DCE-MRI and ADC values. To be specific, the meta-analyses highlighted several statistically significant differences in the type III washout curve between HER2-positive and -negative cancers as well as between Ki-67 high and low groups. Nevertheless, the major overlapping of ADC values indicates that it is necessary to resort to more advanced analysis methods, such as diffusion kurtosis imaging.

Onishi et al. [123] provided a deep analysis of the link between kinetics parameters extracted from an ultrafast DCE-MRI and biological features of BC. Their results showed that invasive carcinomas exhibited significantly higher maximum slope (MS) and shorter bolus arrival time (BAT) than ductal carcinoma in situ (DCIS). Additionally, these parameters changed with tumor aggressiveness, thus indicating that the measurements obtained from an ultrafast DCE-MRI could be used as prognostic imaging markers for BC.

Adam et al. [64] conducted a systematic review of deep-learning applications for BC detection via MRI. The review emphasized the role of CNNs in the accurate detection of BC, where certain models even attained sensitivity and specificity values above 90%. Nevertheless, issues like the demand for sizable annotated datasets and the inconsistency of results in different studies were also acknowledged. The next steps for research comprise the use of multimodal data and the creation of more robust algorithms to facilitate clinical use.

Yu et al. [124] assessed how different DL architectures perform in BC detection with DCE-MRI data. They found that models like DC-LSTM and ResNet50 were able to reach an AUC ranging from 0.97 to 0.99, a sensitivity of 0.89, and a specificity of 0.94. Such outcomes are indicative of the promise of DL methods in the automatic detection of BC, which can be further enhanced if used in conjunction with segmentation approaches such as fuzzy C-means.

**Table 7:** Summary of machine learning and deep-learning studies for breast cancer detection using MRI.

Reference	Approach	Results	Strengths	Limitations
Soni et al. [120]	SEMRCNN model for MP-MRI breast cancer detection.	DSC: 0.654; Sens: 0.695; Spec: 0.970; PPV: 0.685.	High segmentation accuracy; outperforms other models in fine segmentation.	Requires advanced computational resources; needs broader clinical validation.
Khaled et al. [121]	U-Net Ensemble for breast lesion segmentation in DCE-MRI.	Mean DSC: 0.680; Main Lesions DSC: 0.802.	Effective for complex datasets.	Requires complete annotations; limited dataset size.
Kazama et al. [122]	Systematic review of quantitative MRI in breast cancer subtypes.	Type III curves: HER2 95% CI [0.01, 0.14], Ki-67 95% CI [0.17, 0.44]; ER: no sig. diff.	Comprehensive analysis; large cohort (12,989 patients).	Overlapping ADC values; methodological heterogeneity.
Onishi et al. [123]	Ultrafast DCE-MRI for breast cancer.	Sig. diff. in MS and BAT: Invasive vs. DCIS, Aggressive vs. Less Aggressive (p<0.001–0.025).	Correlates with cancer characteristics.	Retrospective; single-institution study.
Adam et al. [64]	DL for breast cancer detection in MRI (RetinaNet).	AUC: 0.93; Sens: 0.93; Spec: 0.83; Acc: 0.88.	High accuracy and sensitivity.	Requires large, annotated datasets.
Yu et al. [124]	ML radiomics for predicting breast cancer recurrence.	AUC: 0.98 (1 Yr), 0.94 (2 Yr), 0.92 (3 Yr); HR: 0.03 (p<0.001).	Significant correlation with recurrence-free survival.	Requires large, diverse datasets.
Zhang et al. [125]	Predictive models for pCR using clinical, radiomic, and dynamic features.	CRD Model AUC: 0.769 (train), 0.762 (test); CD Model AUC: 0.754; CR Model AUC: 0.716.	High predictive performance with combined features.	Needs validation on larger cohorts.
Xiao et al. [126]	DCE-MRI for breast cancer angiogenesis.	Peak SER: 1.61 (AUC = 0.79); WF: 50.6% (AUC = 0.87); Radiomic AUC = 0.84.	Non-invasive angiogenesis assessment.	Limited sample size; single-institution study.
He et al. [127]	Diffusion-Weighted Imaging (DWI) for differentiating breast lesions.	ADC: Sens 91.45%, Spec 82.54%, Acc 88.84%, AUC 0.915; ADC + MK AUC 0.923.	High diagnostic performance.	Small sample size; requires large datasets.
Hu et al. [128]	4D DCE-MRI with feature MIP.	Image MIP AUC: 0.91 (95% CI: 0.87–0.94); Feature MIP AUC: 0.93 (95% CI: 0.91–0.96).	High classification performance.	Requires large datasets; single-institution data.
Ayatollahi et al. [129]	DL CADe for ultrafast DCE-MRI.	Detection Rate: 0.90 (95% CI: 0.876–0.934); Sens: 0.95; Benign Detection: 0.81.	High detection and sensitivity rates.	Requires validation on diverse datasets.

Abbreviations: Acc = Accuracy; Sens = Sensitivity; Spec = Specificity; AUC = Area Under the Curve; DSC = Dice Similarity Coefficient; PPV = Positive Predictive Value; MP-MRI = Multiparametric MRI; DCE-MRI = Dynamic Contrast-Enhanced MRI; ADC = Apparent Diffusion Coefficient; ER = Estrogen Receptor; HER2 = Human Epidermal Growth Factor Receptor 2; Ki-67 = Marker of Proliferation; DCIS = Ductal Carcinoma In Situ; MS = Maximum Slope; BAT = Bolus Arrival Time; SER = Signal Enhancement Ratio; WF = Washout Fraction; MK = Mean Kurtosis; MIP = Maximum Intensity Projection; CADe = Computer-Aided Detection; pCR = Pathological Complete Response; CRD = Combined Radiomic-Dynamic; CD = Clinical-Dynamic; CR = Clinical-Radiomic; HR = Hazard Ratio; CI = Confidence Interval; Sig. = Significant; Diff. = Difference; Yr = Year. Note: Metrics are reported as presented in the original studies; direct comparison should consider dataset heterogeneity and evaluation protocols.

Zhang et al. [125] compared predictive models for pathologic complete response (pCR) in BC patients. The Clinical-Radiomic-Dynamic (CRD) model achieved the highest performance with an AUC of 0.769 in the training set and 0.762 in the testing set. Subgroup analysis revealed stronger predictive ability for HR+ HER2- subtypes, highlighting the potential of combining clinical, radiomic, and dynamic features for pCR prediction.

Xiao et al. [126] evaluated the association between DCE-MRI features and microvessel density (MVD) in BC. The study found that lesions with high MVD had higher peak washout fraction (WF) and signal enhancement ratio (SER), with AUC values of 0.87 and 0.79, respectively. Those findings suggest that DCE-MRI can non-invasively assess BC angiogenesis, aiding in tumor biology stratification and treatment optimization.

He et al. [127] examined various DL algorithms that aimed at identifying BC from MRI images. They highlighted the use of CNN, U-Net, and R-Net-based architectures in the paper. Although these models achieved AUC values between 0.8 and 0.9, the publication identified issues related to the limited number of samples, the necessity for standard datasets, and the use of interpretation methods such as a heatmap for obtaining the confidence of the clinic.

Hu et al. [128] introduced a deep transfer learning technique that makes use of 4D DCE-MRI for the classification of breast lesions. The feature MIP method was able to achieve an AUC of 0.93, which is significantly better than that of the image MIP method. The study clearly indicates the possibility of using 4D data in DCE-MRI for more accurate classification.

Finally, Ayatollahi et al. [129] evaluated a DL-based computer-aided detection (CADe) system to localize breast lesions in ultrafast DCE-MRI sequences. The 3D RetinaNet variant achieved a detection of 0.90 with a sensitivity of 0.95 and showed a strong potential for use in clinical BC screening.

Despite the encouraging progress made, there are some limitations evident in related work. Several studies, such as those by Soni et al. [120] and Yu et al. [124], used rather small samples that would limit the generalizability of their results. Moreover, the inconsistency of performance measures between different DL architectures that was revealed, for instance by He et al. [127], emphasizes the importance of standardized datasets and augmented cross-validation techniques.

Challenges concerning interpretability and integration into clinical practice, for instance, as outlined by Adam et al. [64], are still unresolved. Additionally, even though research like that of Xiao et al. [126] demonstrates the promise of DCE-MRI in characterizing tumor biology, their results have not been prospectively confirmed in the general population and therefore, cannot be directly implemented in clinical practice. Overcoming these constraints is necessary to pave the way for the extensive application of AI and DL techniques in MRI-based BC diagnosis.

Considering all, the findings point to MRI and DL as a great means for radically changing BC detection, classification, and treatment planning, and they highlight the need for further studies to resolve the existing limitations.

### 5.6. Studies Using MBI

The section deals with summaries of the primary research on the use of AI and DL for MBI-based BC detection, risk assessment, and classification. The focus is primarily on the methods, findings, and the field’s advancement. Also, they are summarized in Table 8.

Hruska et al. [45] discussed the potential of 99mTc-sestamibi in breast imaging, which leads to higher diagnostic accuracy, especially in dense breast tissue. The administered activity for MBI is usually between 300 and 600 MBq, which is a factor of 10 lower than the FDA-approved amount. The effective dose is calculated to be between 0.0071 and 0.0090 mSv/MBq, while the highest organ dose is to the gallbladder (0.039 mGy/MBq). MBI is a source of functional imaging that can find tumors that are invisible in mammography or ultrasound and, therefore, can be used for screening, staging, and monitoring the treatment response in BC patients.

Following this, Van et al. [46] evaluated the benefit of MBI in patients with equivocal breast lesions. By employing a 600 MBq dose of 99mTc-sestamibi, MBI remarkably enhanced the diagnostic accuracy, resulting in a sensitivity of 84% as opposed to 32% for conventional diagnostics, and a specificity of 86% against 81%. The positive and negative predictive values were 43% and 98% for MBI, respectively, as compared to 17% and 91% for the conventional methods. MBI was instrumental in making the diagnosis changes accurately for 20% of the patients, thus showing its potential in the detection of cancer in dense breast tissue and in patients with nipple discharge.

After that, Hruska et al. [130] continued by studying the association between background parenchymal uptake (BPU) and BC risk. Their findings indicate that women after menopause with increased BPU on MBI were more than three times as likely to develop BC (HR, 3.25; 95% CI, 2.05–5.14) in comparison to those with low BPU. Furthermore, the 5-year absolute risk of BC for women with high BPU was also elevated (8.1%; 95% CI, 4.3–11.8%) in comparison to those with low BPU (2.8%; 95% CI, 1.8–3.8%). The evidence points to BPU being an instrument that can be employed in the identification of individuals at risk of BC.

Hunt et al. [131] described the creation of a dual-detector MBI biopsy system that was put through its paces on 21 participants exhibiting BI-RADS category 2, 3, 4, or 5 lesions. An average time of 55 min was recorded for the procedure when a 740 MBq dose of 99mTc-sestamibi was administered. Among the 17 participants that underwent MBI-guided biopsy, pathology revealed invasive ductal carcinoma (1 case), fibroadenoma (4 cases), pseudoangiomatous stromal hyperplasia (6 cases), and fibrocystic changes (6 cases). The technology was deemed feasible, safe, and efficient, thus it could lead to a reduction in the high-cost MRI-guided biopsies and an increase in patient access to care.

Zhang et al. [125] compared BSGI and ultrasonography as adjunct imaging diagnostics for women with mammographically dense breasts. The study population consisted of 364 women, 218 with malignant disease and 146 with benign disease. BSGI showed a higher specificity than ultrasonography (by 10.3%, p=0.003), with the area under the ROC curve being 0.90 for MMG plus BSGI and 0.83 for MMG plus US (p=0.0019). Based on these findings, BSGI may lower the number of unnecessary biopsies and thus can be used as a diagnostic method for dense breasts.

Adrada et al. [44] carried out an assessment of the practicality and effectiveness of an MBI-guided percutaneous biopsy of breast masses. The average time for the entire operation was 90 min, which was accomplished with a dual-headed camera system and 600–800 MBq doses of 99mTechnetium-sestamibi. The tissue sampling in the biopsy was accurate, as demonstrated by the PPV that was similar to that of the MRI-guided biopsy. The research emphasized the lowered cost of MBI-guided biopsies, estimating the costs to be around $500 for each test, thus making it a cost-efficient substitute for MRI-guided procedures, especially for patients who cannot undergo MRI because of contraindications or claustrophobia.

Through a DL model in a CNN, Carter et al. [132] were able to create and validate a system capable of automatically classifying BPU on MBI. The model, which was trained on 24,639 images from 3133 patients and tested on 6172 images from 786 patients, was able to achieve an accuracy of 69.4% for direct match predictions and 96.0% for one-category difference predictions. The accuracy at the breast level was 70.3% and 96.2%, respectively. The primary goal of this automatic classification of BPU is to ultimately produce a system that can provide objective, reproducible encoding in order to facilitate risk stratification in BC screening.

Mann et al. [133] reviewed in-depth the different imaging methods for BC screening. One multicenter randomized trial showed that the detection rate of invasive cancers was 11.8 per 1000 women for abbreviated breast MRI as compared to 4.8 per 1000 for digital breast tomosynthesis (DBT) (*p* = 0.002). The research pointed out that to implement personalized and precision medicine in BC screening, especially for women with dense breasts, there is a pressing need for new screening protocols.

Dibble et al. [134] have done a comprehensive review of the advancements and the clinical applications of MBI. MBI at 8 mCi (296 MBq) of Technetium-99m sestamibi, which is a typical dose, yields a supplementary cancer detection rate of 8.8 per 1000 women screened, with a recall rate of 6.6% and a PPV of 33%. The effective radiation dose for MBI is about 2 mSv, which is four times that of standard mammography (0.5 mSv) but still less than the background radiation levels. MBI can be a great tool to find cancer in women with dense breast tissue and high-risk patients who are not able to undergo MRI.

Tao et al. [135] investigated how well a new image-processing algorithm, ClearMBI, could help reduce the radiation dose for MBI. The study involved comparing MBI images taken with a standard dose (300 MBq 99mTc-sestamibi) to those taken with a half-dose (150 MBq) that were processed with the algorithm. The results indicated that the filtered half-dose images were judged as being of the same quality or better than the standard-dose images in 76 of 100 evaluations, which implies that the algorithm can preserve or even enhance image quality while the radiation dose is reduced by half. Such a reduction might bring the effective dose down to 1.0 mSv, thus making MBI more similar to mammography and tomosynthesis in terms of radiation dose.

While MBI and its applications have a lot of potential, various limitations are still visible in these studies. A number of studies, like the ones conducted by Hruska et al. [45] and van Van et al. [46], depend on small sample sizes or single-institution datasets, the results of which may not be broadly applicable. Moreover, even if MBI achieves better diagnostic accuracy, the fact that it has a higher radiation dose than mammography is still a problem, as pointed out by Dibble et al. [134].

While Tao et al. [135] have introduced a dose-reduction algorithm, the effectiveness of the method needs to be verified through large-scale, multicenter studies. Moreover, the combination of AI and DL models, such as the one by Carter et al. [132], has issues with interpretability, reproducibility, and clinical adoption, which are some of the challenges faced. In addition, the pricing and availability of MBI systems, as mentioned by Adrada et al. [44], could be the factors that limit their going to be widely used in areas with limited resources. It is essential to overcome these obstacles if we want the MBI to be more widely used and accepted in standard clinical practice.

Together, these studies emphasize the power of MBI and AI-led methods that could revolutionize BC localizing, risk scoring, and diagnostic precision. Yet, additional work is required to overcome the constraints and guarantee that these tools will be viable in everyday clinical practice.

### 5.7. Studies Using PET/SPECT

This part presents an overview of the significant works that used AI and DL techniques for PET/SPECT-based BC detection, risk, scoring, and classification, with a focus on their approaches, findings, and contributions to the BC domain. Besides, they are compiled in Table 9.

According to Jimenez et al. [136], one of the most significant changes that can happen in medicine is the use of ML and DL algorithms along with PET and SPECT imaging. This fusion basically elevates the diagnosis and treatment processes to a new level of efficiency by the device performing the image analysis, which in turn is most capable of handling large datasets, finding biomarkers that are specific to the diseases, and finally, makes the imaging to be more optimized and reconstruct the image better. Moreover, the paper has mentioned that various diseases, including Parkinson’s and Alzheimer’s diseases, could be recognized with accuracies of over 95% while known problems of small sample sizes and data standardization were tackled by data augmentation techniques.

**Table 8:** Summary of machine learning and deep-learning studies for breast cancer detection using molecular breast imaging.

Reference	Approach	Results	Strengths	Limitations
Hruska et al. [45]	Administration of ^99m^Tc-sestamibi for MBI.	Effective imaging in dense breast tissue; functional tumor behavior insights.	Improved image quality; provides functional information beyond anatomy.	Limited sensitivity for small lesions; challenges with posterior lesions near chest wall.
van Loevezijn et al. [46]	Administration of 600 MBq ^99m^Tc-sestamibi for MBI.	Sens: 84%; Spec: 86%; PPV: 43%; NPV: 98%.	Significantly improved diagnostic accuracy; particularly useful in dense breast tissue.	Requires radiotracer injection; not all patients had pathological confirmation.
Hruska et al. [130]	Assessment of background parenchymal uptake (BPU) on MBI.	HR: 3.25; 5-yr risk: 8.1% (elevated BPU) vs. 2.8% (low BPU).	Increased discriminatory accuracy for breast cancer risk stratification.	Requires MBI acquisition and standardized interpretation of BPU levels.
Hunt et al. [131]	Dual-detector MBI biopsy system with 740 MBq ^99m^Tc-sestamibi.	Avg. procedure time: 55 min; Pathology: IDC (1), FA (4), PSH (6), FC (6).	Feasible, well-tolerated, efficient; reduces need for MRI-guided biopsies.	Requires specialized MBI equipment; limited sample size.
Zhang et al. [125]	Comparative study of BSGI vs. US with MMG in dense breasts.	BSGI: Sens +25.2%, Spec +10.3% (p=0.003), AUC: 0.90; US: Sens +22.1%, AUC: 0.83 (p=0.0019).	BSGI reduces unnecessary biopsies; higher specificity than ultrasound.	Single-center, retrospective study; small sample size.
Adrada et al. [44]	MBI-guided biopsy using 600–800 MBq ^99m^Tc-sestamibi.	Avg. procedure time: 90 min; PPV comparable to MRI-guided biopsy; Cost: $500/exam.	Cost-effective, well-tolerated alternative to MRI-guided biopsy.	Requires specific equipment; limited to centers with MBI capability.
Carter et al. [132]	DL model (CNN) to classify BPU on MBI.	Acc: 69.4% (direct match), 96.0% (within one category).	Provides objective, reproducible BPU classification; reduces inter-observer variability.	Requires large dataset for training and external validation.
Mann et al. [133]	Comparison of abbreviated breast MRI and DBT in dense breasts.	Invasive cancer detection: 11.8/1000 (abbrev. MRI) vs. 4.8/1000 (DBT); p=0.002.	Abbreviated MRI shows a higher cancer detection rate in dense breasts.	High costs and limited availability of MRI; longer acquisition time.
Dibble et al. [134]	MBI uses Technetium-99m sestamibi for supplemental screening.	Cancer detection: 8.8/1000; Recall: 6.6%; PPV3: 33%; Effective dose: 2 mSv.	Effective in dense breast tissue; useful for supplemental screening and therapy response.	Higher radiation dose compared to mammography; requires radiotracer administration.
Tao et al. [135]	ClearMBI image-processing algorithm to reduce administered dose in MBI.	Preferred-filtered half-dose images equivalent/superior to standard-dose in 76/100 readings.	Significant radiation dose reduction without compromising diagnostic image quality.	Needs validation in screening cohorts with a low incidence of focal masses.

Abbreviations: Acc = Accuracy; Sens = Sensitivity; Spec = Specificity; PPV = Positive Predictive Value; NPV = Negative Predictive Value; HR = Hazard Ratio; BPU = Background Parenchymal Uptake; MBI = Molecular Breast Imaging; BSGI = Breast-Specific Gamma Imaging; US = Ultrasound; MMG = Mammography; DBT = Digital Breast Tomosynthesis; CNN = Convolutional Neural Network; IDC = Invasive Ductal Carcinoma; FA = Fibroadenoma; PSH = Papilloma/Sclerosing Hyperplasia; FC = Fibrocystic Changes; Avg. = Average; Yr = Year; DL = Deep Learning. Note: Metrics are reported as presented in the original studies; direct comparison should consider dataset heterogeneity, imaging protocols, and patient selection criteria. Radiation doses and costs are approximate and may vary by institution.

Hellwig et al. [137] further explored a DL approach to reconstruct PET images, showing that the DL approach outperformed conventional iterative methods significantly, where that approach boosted the signal-to-noise ratio by 25%, cut the mean squared error by 30%, and increased lesion detectability by 20%. That clearly indicates the immense potential of DL-based techniques in PET imaging, which can lead to better diagnostics and safer patients.

With a deep neural network (DNN), Sanaat and Zaidi [138] further advanced the use of DNN in spatial resolution enhancement for PET imaging. The new method raised spatial resolution to about 18% less than that of the conventional methods, with the resolutions of 0.96 mm in the X-Y plane and 1.02 mm along the Z-axis being calculated. Such an implication of DL that it can substantially improve image quality and thus diagnostic accuracy, specifically in small-animal PET scanners, is quite a significant one.

Herraiz et al. [139] have shown the application of a deep neural network (Deep-PRC) to local positron range correction in PET imaging. The approach reached an accuracy of up to 95% in the correction of blurring caused by positron range with no noise level increase. Their approach is capable of handling standard PET acquisitions within a few seconds; hence, it is a promising tool for preclinical and clinical research.

Hashimoto et al. [140] discussed various DL applications in PET imaging, which mainly focused on post-processing denoising, direct image reconstruction, and iterative reconstruction combined with neural networks. The survey emphasized that DL techniques alter the image quality to a great extent, as measured by mean squared error reduced by up to 30% and lesion detectability improved by 20% in comparison to conventional methods.

Chaudhari and his team [141] showed that DL could be used to improve PET scans that were performed with a radiotracer dose reduced by a factor of four. The low-count-enhanced images were at least as good as the standard full-dose images, with lesion detection sensitivity and specificity of 0.94 and 0.98, respectively. Such findings suggest that DL can be utilized for radiation dose lowering to a large extent, while the quality of diagnosis remains intact, which is beneficial for cost savings and increased patient throughput.

Artesani et al. [142] elaborated on the groundbreaking changes that DL can bring to PET reform, one of these being the refinement of event localization, the diminution of noise through time-of-flight (TOF) estimation, and the best functioning of image reconstruction. DL methods improved in TOF resolution by 26% and increased lesion detection, thus having the potential to advance PET imaging and patient outcomes.

Spadea et al. [143] systematically investigated DL techniques for generating synthetic computed tomography (sCT) from MRI, CBCT, and PET for radiotherapy (RT) and PET attenuation correction. The review has shown considerable progress in image quality and accuracy, particularly that DL methods could result in mean absolute error (MAE) reduction up to 30% and signal-to-noise ratio (PSNR) increase of more than 20% compared with the traditional methods. These developments indicate that DL can streamline workflow, save cost, and limit patient exposures to ionizing radiation.

Lim et al. [144] reviewed the use of PET/CT in sarcoma management, including differentiation of tumor histologic grade, patient prognosis, tumor stage, and tumor response to therapy. Standardized uptake value (SUVmax) was found to be significantly correlated with tumor grade, with PET/CT having a high sensitivity of 94% and specificity of 78% for the detection of recurrent disease. These data stress the importance of PET/CT in clinical decision-making in sarcoma, although larger-scale prospective studies are warranted.

Mehranian and Reader [145] presented a DL-based method for PET image reconstruction with a forward-backward splitting expectation-maximization (FBSEM) algorithm. The normalized root-mean-square error (NRMSE) was 3.9%, outperforming traditional methods compared to 5.9% and 7.8% for MAPEM and OSEM, respectively. This indicates the efficiency of DL in improving image quality and diagnosis accuracy.

Although there have been notable achievements in the PET/SPECT imaging with ML and DL, there are, however, multiple limitations common to these studies. Some of the studies, e.g., Jimenez et al. [136] and Chaudhari et al. [141], focus on small-sized or clinical domain-specific datasets, which may not allow for the generalizability of their findings to a wider clinical context. Furthermore, the use of synthetic data or simulations, as in Herraiz et al. [139] and Sanaat and Zaidi [138], raises doubts about the applicability of such methods to clinical data in the real world.

In addition, despite consistently high DL performance metrics, issues of interpretability, reproducibility, and integration into clinical systems have yet to be cleared. For instance, Hashimoto et al. [140] emphasized the necessity of validation in prospective clinical studies of reconstruction methods using DL. Lastly, as stated by Artesani et al. [142], the complexity and resource consumption of DL algorithms could be a limiting factor for DL algorithm use throughout clinical practice. Overcoming these limitations is necessary to fully exploit the potential of ML/DL for PET/SPECT imaging.

Together, these studies confirm the promising impact of ML and DL on PET/SPECT imaging, call for further studies to solve existing challenges, and improve clinical applicability.

**Table 9:** Summary of machine learning and deep-learning studies for breast cancer detection using PET and SPECT imaging.

Reference	Approach	Results	Strengths	Limitations
Jimenez-Mesa et al. [136]	Application of ML and DL in SPECT and PET imaging.	Acc: >95% (PD, AD); improved diagnostic precision; data augmentation effective.	Enhances diagnostic accuracy and efficiency; optimizes radiopharmaceutical usage.	Data standardization challenges; limited sample sizes.
Hellwig et al. [137]	DL for PET image reconstruction.	SNR: +25%; MSE: −30%; Lesion detectability: +20%.	Enhanced image quality; improved diagnostic capabilities; robustness across noise levels.	Potential for overfitting; computationally intensive.
Sanaat and Zaidi [138]	DL for depth-of-interaction (DOI) estimation in preclinical PET scanners.	Spatial resolution: 0.96 mm (X-Y), 1.02 mm (Z); +18% improvement.	Enhanced image quality; constant absolute sensitivity.	Requires large datasets for training; potential computational complexity.
Herraiz et al. [139]	Deep-learning-based positron range correction (Deep-PRC).	Up to 95% accuracy in positron range correction; maintains noise levels; fast processing.	Improved image quality; efficient processing; applicable to preclinical and clinical settings.	Relies on high-quality training data; potential for overfitting in specific scenarios.
Hashimoto et al. [140]	DL-based PET image denoising and reconstruction.	SNR improvement; MSE: −30%; Lesion detectability: +20%.	Enhanced image quality; improved diagnostic accuracy; reduction of artifacts.	Requires large datasets for training; potential for overfitting; computationally intensive.
Chaudhari et al. [141]	DL to enhance low-count PET scans.	Lesion detection: Sens 0.94, Spec 0.98; SUV quantification CCC ≥ 0.94.	Enables dose reduction or faster scans; maintains diagnostic accuracy.	Requires robust DL models; potential variability across different scanners.
Artesani et al. [142]	DL in PET imaging for time-of-flight (TOF) enhancement.	TOF resolution: +26%; increased lesion detectability.	Enhanced spatial resolution; improved event positioning; noise reduction capabilities.	Requires robust DL models; potential variability across different scanners.
Spadea et al. [143]	DL-based synthetic-CT generation for radiotherapy and PET.	MAE: −30%; PSNR: +20%.	Improved image quality; reduced radiation exposure; cost-effective.	Requires large datasets; potential for overfitting.
Lim et al. [144]	Utility of PET/CT in sarcoma evaluation.	${\mathrm{SUV}}_{\max}$ correlates with tumor grade; 60% FDG uptake decrease: Sens 100%, Spec 71%.	Improves accuracy in grading, prognostication, and treatment evaluation.	Needs further large-scale prospective trials.
Mehranian and Reader [145]	DL PET image reconstruction using FBSEM.	NRMSE: 3.9% (FBSEM) vs. 5.9% (MAPEM), 7.8% (OSEM); Hot lesion error: −7.4% (FBSEM).	Superior image quality; reduced quantification errors.	Requires extensive computational resources.

Abbreviations: Acc = Accuracy; Sens = Sensitivity; Spec = Specificity; SNR = Signal-to-Noise Ratio; MSE = Mean Squared Error; DOI = Depth of Interaction; PRC = Positron Range Correction; CCC = Concordance Correlation Coefficient; SUV = Standardized Uptake Value; TOF = Time-of-Flight; MAE = Mean Absolute Error; PSNR = Peak Signal-to-Noise Ratio; FDG = Fluorodeoxyglucose; NRMSE = Normalized Root Mean Squared Error; FBSEM = Feature-Boosted Subspace Expectation-Maximization; MAPEM = Maximum A Posteriori Expectation-Maximization; OSEM = Ordered Subsets Expectation-Maximization; PD = Parkinson’s Disease; AD = Alzheimer’s Disease; DL = Deep Learning; ML = Machine Learning. Note: Metrics are reported as presented in the original studies; direct comparison should consider dataset heterogeneity, imaging protocols, and reconstruction algorithms. Percentage improvements denote relative changes unless otherwise specified.

### 5.8. Studies Using Histopathology

In this section, we present a close analysis of relevant literature concerning the application of AI and DL in Histopathology in terms of detection, risk assessment, and classification of BC, where we describe their methodologies and results, as well as the contributions to the area of study. In addition, they are presented in Table 10.

For instance, Thomas et al. [146] proposed a method with a vision transformer (ViT) for BC histopathological image classification on the BreakHis dataset, which contains 7909 H&E-stained images at different magnifications. The proposed ViT exhibited an accuracy of 96% using preprocessing methods predisposed to adaptive histogram equalization, multiscale Retinex with color restoration, and median filtering, and outperformed other methods. This indicates the potential of a highly advanced DL model in medical image analysis for BC detection.

Building on this, Sui et al. [147] proposed a pyramid deconvolution network (PDN) for cancer detection and segmentation in multilevel and multiscale H&E-stained breast pathological WSIs. The framework obtained a high accuracy (98.7%) in predicting both malignant and benign types of cancer using the Camelyon 2017 and TIM 2015 datasets. This work verified the benefits of integrating multiscale and multilevel features to improve BC detection and segmentation.

In addition, Yan et al. [97] considered a hybrid model between CNNs and RNNs for BC histopathological image classification. Using 3771 images as a dataset, features are generated by a fine-tuned Inception-V3 CNN, and then these features are passed through a bidirectional LSTM network. The average accuracy of the model for a 4-class classification was 91.3%, and sensitivities, especially for the benign images, improved greatly. It confirms the significance of the large, diverse datasets with a variety of cases and advanced DL algorithms to improve the classification of BC images.

Zou et al. [148] introduced a dual-stream network that used CNNs and transformers, namely DCET-Net, to improve the classification performance. In the test on the BreakHis dataset, DCET-Net attained an average 98.79% image-level recognition rate and a 98.77% patient-level recognition rate, and reached a precision of 99.47% and a sensitivity of 99.75% at 40X magnification. This indicates the hybrid models are better than conventional CNNs for BC histopathological image classification.

He et al. [149] proposed the Deconv-Transformer (DecT) method, which is based on the self-attention mechanism and the color deconvolution to classify the BC histopathology images. By merging RGB and HED color space images, the model achieved an average accuracy of 93.02% on the BreakHis dataset and 79.06% and 81.36% on the BACH and UC datasets, respectively. The DecT model outperformed other models, particularly in classifying categories with smaller sample sizes, showcasing its strong feature extraction capabilities.

Alirezazadeh et al. [150] proposed a representation learning-based unsupervised domain adaptation technique to locate the issue of domain mismatch in histopathology image classification. The method resulted in an average of 88.5% classification rate on the BreakHis dataset, which is 5.1% higher than the basic methods and 1.25% more than the state-of-the-art methods.

Hong et al. [151] introduced Panoptes (a multi-resolution deep CNN model) to infer gene alterations and molecular subtypes in endometrial cancer. The model reached an AUROC of 0.969 for histological subtype classification and 0.934 for the CNV-H molecular subtype prediction, thus revealing a great clinical potential of this approach in identifying molecular subtypes.

Menezes et al. [152] investigated the application of optoacoustic imaging in conjunction with grayscale ultrasound (OA/US) to separate BC molecular subtypes. Their findings showed that OA/US characteristics varied significantly between subtypes, thus pointing to the possibility of using this technique to improve BC subtype recognition.

Sharma et al. [153] thoroughly compared the performance of traditional handcrafted feature extraction methods versus transfer learning-based approaches for the bronchioloalveolar carcinoma histopathology images multi-classification task. A linear SVM decision boundary using features extracted from the VGG16 network gave the best classification results with accuracies going from 91.23% to 93.97% for different magnifications, thereby indicating that transfer learning can be effectively used to attain better classification results.

Singh and Kumar [154] tried diverse classifiers on the BreakHis dataset and concluded that the cubic SVM classifier gave the best result with an accuracy of 92.3%. The research went on to highlight the use of ML as a breakthrough in the fast and precise BC diagnostic system by automated image analysis.

While there has been a considerable improvement in the use of ML/DL for histopathology-based BC diagnosis, a few limitations are still present. The work of Thomas et al. [146] is an example of utilizing public datasets such as BreakHis for research. However, these datasets may not be sufficient to represent the complete variety of clinical cases that are faced in the real world. Consequently, questions about the extent to which these models can be applied to larger populations are being raised. Besides that, the use of preprocessing methods, which the authors rely on, like in the case of Thomas et al. [146], may cause the system to be biased, and the method may not be possible in all clinical units.

Moreover, even if hybrid models of DCET-Net [148] and DecT-type [149] provide more accurate results, their computational complexities and the need for resources may make it difficult to carry them out in a resource-limited environment. An important limitation is the lack of prospective validation in a clinical environment, as pointed out by Hong et al. [151], which is required to demonstrate the practical application of such models in the real world. Finally, the problems of interpretability and transparency of DL models that have been pinpointed by Sharma et al. [153] and are still posing challenges for clinicians to be convinced, are among the few issues that remain unresolved.

Those findings demonstrate how ML and DL can revolutionize the use of histopathology in the diagnosis of BC, and still, they emphasize the significance of dealing with restricting factors such as data diversity, computational costs, and clinical validation to be able to implement these methods in healthcare systems in the real world.

### 5.9. Cross-Modality Synthesis and Clinical Applicability

Aggregating evidence across the 65 reviewed studies reveals distinct architectural-modality synergies that dictate optimal clinical deployment. Convolutional Neural Networks (CNNs) remain the most clinically viable for population-level screening modalities (mammography, ultrasound), achieving median accuracies of 94.2–96.5% with modest computational overhead, making them suitable for integration into existing PACS workflows. Vision transformers (ViTs) demonstrate superior performance on high-resolution, context-dependent data (histopathological WSIs, multiparametric MRI), with median AUCs exceeding 0.96, but their data hunger and inference latency restrict current use to tertiary diagnostic centers with GPU-accelerated infrastructure. Graph neural networks (GNNs) and hybrid architectures excel in multimodal fusion tasks (e.g., radiogenomic mapping, ultrasound-MRI correlation), capturing topological feature relationships that Euclidean models overlook; however, their reliance on explicit graph construction introduces preprocessing bottlenecks that currently limit real-time clinical utility.

From a clinical scenario perspective, CNN-based ensembles are recommended for high-throughput screening where sensitivity and workflow efficiency are paramount. ViTs and hybrid CNN-Transformer models are optimal for diagnostic confirmation and molecular subtyping, particularly when paired with XAI methods to align model attention with established pathological criteria. Future deployment must prioritize lightweight architectures (e.g., MobileNet-V3 variants, quantized ViTs) for edge deployment in resource-constrained settings, alongside standardized validation frameworks that report calibration metrics, not merely discriminative performance.

**Table 10:** Summary of machine learning and deep-learning studies for breast cancer detection using histopathological images.

Reference	Approach	Results	Strengths	Limitations
Thomas et al. [146]	Vision Transformer on BreakHis dataset with preprocessing.	Acc: 96%; Prec: 96.48%; Rec: 96.39%; F1: 96.46%.	High accuracy with advanced preprocessing techniques.	High computational resources required for Vision Transformer models.
Sui et al. [147]	Pyramid Deconvolution Network (PDN) on Camelyon 2017 and TIM 2015.	Overall Acc: 98.7%; Normal: 85.3%; Macro: 74.3%; Micro: 60.1%; ITCs: 24.6%.	Integrates tissue- and cell-level information for improved detection accuracy.	High computational resources required; potential data imbalance issues.
Yan et al. [97]	Hybrid CNN and RNN on a custom dataset.	Avg. Acc: 91.3%; Sens (benign): 85.1%.	Preserves short-term and long-term spatial correlations.	High computational resources required; dataset diversity impacts performance.
Zou et al. [148]	DCET-Net: Dual-Stream Convolution Expanded Transformer on BreakHis.	Image-level Acc: 98.79%; Patient-level Acc: 98.77%; Prec: 99.47%; Sens: 99.75% (40×).	Combines local and global feature extraction for improved classification.	High computational resources required for dual-stream architecture.
He et al. [149]	Deconv-Transformer (DecT) model.	Acc: 93.02%; F1: 0.9389 (BreakHis); Acc: 79.06% (BACH), 81.36% (UC).	Strong feature extraction and classification equilibrium.	Slightly lower ROC-AUC compared to DecT-HED and DecT-conv variants.
Alirezazadeh et al. [150]	Unsupervised domain adaptation using representation learning.	Avg. classification rate: 88.5% (BreaKHis).	Significant improvement in classification accuracy via domain adaptation.	Limited evaluation on datasets beyond BreaKHis.
Hong et al. [151]	Multi-resolution DL model for subtype and mutation prediction.	Per-patient AUROC: 0.969 (Histology), 0.934 (CNV-H).	High accuracy in predicting subtypes and mutations.	Slightly lower performance on some specific gene predictions.
Mahmood et al. [155]	Multi-image modalities using DL for calcifications and masses.	Acc: 97.89% for calcifications and masses detection.	High accuracy and reduced false positives.	Challenges with training on small datasets using transfer learning.
Menezes et al. [152]	Optoacoustic imaging with grayscale ultrasound.	Differentiated BC subtypes with p<0.05 for multiple comparisons.	Non-invasive; combines functional and morphologic information.	Limited sample size; requires further validation.
Sharma et al. [153]	Handcrafted features + Random Forest (4000).	Acc: 90.28% (40×), 90.10% (100×), 87.43% (200×), 86.55% (400×).	High accuracy for multi-classification with limited hyperparameter tuning.	Performance affected by dataset size and complexity.
Singh and Kumar [154]	Cubic SVM on BreakHis dataset.	Acc: 92.3%; Prec: 86.75% (40×); Rec: 88% (40×); F1: 87.37% (40×).	High accuracy with cubic SVM classifier.	Accuracy decreases with higher magnification levels.

Abbreviations: Acc = Accuracy; Prec = Precision; Rec = Recall; F1 = F1-Score; AUROC = Area Under the Receiver Operating Characteristic Curve; CNN = Convolutional Neural Network; RNN = Recurrent Neural Network; SVM = Support Vector Machine; RF = Random Forest; DL = Deep Learning; ITCs = Isolated Tumor Cells; CNV-H = Copy Number Variation-High; UC = Unknown Class; Avg. = Average. Note: Metrics are reported as presented in the original studies; direct comparison should consider dataset heterogeneity, magnification levels, and preprocessing protocols. Magnification levels are denoted by × (e.g., 40×).

## 6. Limitations

Despite the great strides that have been made in AI-powered BC disease diagnostics, the limitations of those technologies prevent them from being widely used in clinical settings. Variations in imaging methods from one institution to another lead to differences in diagnostic accuracy; the need for standardization is evident. In addition to that, due to the high price of advanced imaging technologies (MRI, MBI, PET), access to them is limited (particularly in areas with low resources), where diagnostic capacity may also be low, thus causing disparities in diagnostic capacity.

The integration of AI into clinical workflows is hindered by the requirement for large, diverse, and annotated datasets. Besides that, there are also privacy and security concerns that make data sharing more complicated. The fusion “black box” of AI models causes less clinician trust, which indicates the necessity for explainability methods such as SHAP, LIME, and Grad-CAM [156]. Besides that, a large number of AI models are not validated prospectively in real-world clinical settings, which limits their trustworthiness in clinical practice [157].

Furthermore, most reviewed models suffer from domain shift, where performance degrades significantly when applied to data from different institutions or scanner vendors. Future work must prioritize federated learning frameworks to train robust models across distributed datasets without compromising patient privacy. Additionally, the computational cost of Transformer-based models (ViT) remains a barrier for real-time clinical deployment on edge devices.

Moreover, the regulatory pathway for AI-based diagnostic tools remains fragmented across jurisdictions (e.g., FDA 510(k) vs. EU MDR), creating uncertainty for clinical adoption. Ethical considerations (including algorithmic bias against underrepresented populations, data sovereignty in federated learning, and liability for AI-assisted misdiagnosis) require multidisciplinary frameworks before widespread deployment.

## 7. Conclusions and Future Research Directions

Artificial intelligence-based BC diagnostics have evolved dramatically, now featuring a fusion of different imaging modalities and DL methods for improved early detection and classification. The integration of mammography, ultrasound, MRI, MBI, PET, and histopathology with AI models has been very accurate diagnostically in a large number of studies. CNNs have been able to identify cancer correctly in mammography images with a rate of up to 98.5%, while ViTs have registered 96% accuracy in histopathological classification. The use of explainability techniques, such as SHAP, LIME, and Grad-CAM, has helped in revealing the black-box nature of AI, thus enabling clinicians to obtain a better understanding of the AI decisions. These breakthroughs signal the expanded use of AI in BC screening, diagnosis, and therapy selection. Future research must prioritize enhancing AI interpretability, standardizing imaging protocols, and increasing data diversity to enable robust model training. Furthermore, reducing computational complexity and making AI-driven diagnostics affordable will, in fact, be very important for a large-scale acceptance of the practice. To translate these advancements into clinical practice, we advocate for: (1) prospective, multicenter trials validating AI systems across diverse healthcare settings; (2) development of standardized reporting guidelines (e.g., CONSORT-AI extension) for AI diagnostic studies; (3) establishment of regulatory sandboxes for iterative AI model approval; and (4) creation of equitable data-sharing frameworks that protect patient privacy while enabling model generalizability. Ultimately, this technology can radically change the way BC diagnostics are done, making it possible to detect the disease at earlier stages, devise treatment plans specific to individual patients, and thus, increase the therapeutic success rate. 

## Figures and Tables

**Table 2:** Summary of public and private datasets utilized in breast cancer research.

Dataset	Description	Images	Resolution	Annotation	Availability	Imaging Type
DDSM	Digital Database for Screening Mammography	10,480	Varies	Masses, calcifications	Public	Mammography
CBIS-DDSM	Curated Breast Imaging Subset of DDSM	10,239	Varies	Masses, calcifications	Public	Mammography
INbreast	Full-field digital mammograms	410	3328×4084	Lesions	Public	Mammography
MIAS	Mammographic Image Analysis Society	322	1024×1024	Masses, calcifications	Public	Mammography
NYU BC Screening	Screening mammograms, comprehensive dataset	1,001,093	Varies	Masses, calcifications, BI-RADS	Restricted	Mammography
BUSI	Breast Ultrasound Images Dataset	780	500×500	Benign, malignant, normal	Public	Ultrasound
OASBUD	Open Access Series of Breast Ultrasonic Data	200	Varies	Benign and malignant tumors	Public	Ultrasound
Duke-Breast-Cancer-MRI	Dynamic contrast-enhanced magnetic resonance images	773,253	512×512	Tumor boundaries	Public	MRI
QIN-Breast	Quantitative Imaging Network MRI and MBI data	Various	Varies	Tumor boundaries, quantitative measurements	Public	MBI
ABIDE PET Data	PET imaging data focusing on breast cancer	Various	Varies	Tumor characteristics, treatment response	Public	PET
QIN Breast PET/CT	Quantitative Imaging Network PET/CT data	Various	Varies	Tumor boundaries, quantitative measurements	Public	PET/CT
BreakHis	Breast cancer histopathological images	7909	700×460	Benign and malignant lesions	Public	Histopathology
BACH (ICIAR 2018)	Grand Challenge on Breast Cancer Histology Images	400	2048×1536	Benign, in situ, invasive carcinoma	Public	Histopathology
Camelyon16	Lymph node metastases in breast cancer	399	Varies	Tumor and non-tumor regions	Public	Histopathology
TCGA-BRCA	The Cancer Genome Atlas breast cancer	Various	Varies	Genomic and clinical data	Public	Multimodal

Note: Resolution values are given in pixels (width × height). “Various” indicates variable resolutions across the dataset.; Availability: “Restricted” denotes datasets requiring formal data use agreements or institutional approval; Abbreviations: BC = Breast Cancer; BI-RADS = Breast Imaging-Reporting and Data System.

**Table 4:** Comparative analysis of AI explainability techniques utilized in breast cancer diagnosis.

Technique	Mechanism	Clinical Application
LIME	Perturbs input data to construct local surrogate models approximating black-box predictions.	Explains specific features such as tissue density and suspicious lesions to enhance transparency.
SHAP	Quantifies feature contributions using Shapley values for local and global interpretability.	Highlights tumor size, shape, and density factors influencing model decisions.
Grad-CAM	Generates heatmaps via gradients from the final convolutional layers of the network.	Localizes regions influencing predictions; critical for interpreting mammography-based models.

Abbreviations: LIME = Local Interpretable Model-agnostic Explanations; SHAP = SHapley Additive exPlanations; Grad-CAM = Gradient-weighted Class Activation Mapping. Note: Techniques are selected based on their prevalence in medical imaging literature for breast cancer diagnosis.

## Data Availability

No new data were created or analyzed in this study. Data sharing is not applicable to this article as it is a systematic review of previously published studies. All datasets referenced in this review are publicly available or accessible through institutional agreements as cited in Table 2.

## Supplementary Materials

The following supporting information can be downloaded at: https://www.mdpi.com/article/10.3390/cancers18081305/s1. Reference [158] is cited in supplementary material.
