# Supplementary material for: AI-Driven Breast Cancer Diagnosis: A Systematic Review of Imaging Modalities, Deep Learning, and Explainability
Source: Cancers (Basel). 2026 Apr 20;18(8):1305. doi: 10.3390/cancers18081305 (PMC13114346; doi:10.3390/cancers18081305)
Supplement: Supplementary file 1 [file cancers-18-01305-s001.zip › cancers-4224141-supplementary.pdf]

PRISMA 2020 Checklist

Section and Topic	Item #	Checklist item	Location where item is reported
**TITLE**			
Title	1	Identify the report as a systematic review.	Manuscript Title: "AI-Driven Breast Cancer Diagnosis: A Systematic Review of Imaging Modalities, Deep Learning, and Explainability"
**ABSTRACT**			
Abstract	2	See the PRISMA 2020 for Abstracts checklist.	Abstract section (lines 1-19); structured summary of background, methods, results, conclusions
**INTRODUCTION**			
Rationale	3	Describe the rationale for the review in the context of existing knowledge.	Section 1, Introduction (lines 22-49): Discusses global burden of breast cancer, evolution of imaging/AI, and need for comprehensive synthesis
Objectives	4	Provide an explicit statement of the objective(s) or question(s) the review addresses.	Section 1 (lines 49-61); Abstract (lines 3-12): To review AI/DL advancements across imaging modalities for BC diagnosis (2018-2024)
**METHODS**			
Eligibility criteria	5	Specify the inclusion and exclusion criteria for the review and how studies were grouped for the syntheses.	Section 5.1 (lines 538-547): Supervised learning only; published ≥2018; peer-reviewed research/conference papers; empirical results required; grouped by imaging modality
Information sources	6	Specify all databases, registers, websites, organisations, reference lists and other sources searched or consulted to identify studies. Specify the date when each source was last searched or consulted.	Section 5.1 "PRISMA Compliance" (lines 548-555): PubMed, IEEE Xplore, Scopus, Web of Science, Google Scholar, SpringerLink, arXiv, Wiley Online Library; search period: 2018-2024
Search strategy	7	Present the full search strategies for all databases, registers and websites, including any filters and limits used.	Section 5.1 (lines 552-555): Keywords included "breast cancer", "AI diagnosis", "explainable AI", "deep learning", "machine learning", "radiology AI", "medical imaging AI", "computer-aided diagnosis"; filters: publication year ≥2018, English language
Selection process	8	Specify the methods used to decide whether a study met the inclusion criteria of the review, including how many reviewers screened each record and each report retrieved, whether they worked independently, and if applicable, details of automation tools used in the process.	Section 5.1 (lines 554-556): Duplicates removed using automation tools; studies screened against predefined eligibility criteria by authors; consensus reached through discussion
Data collection process	9	Specify the methods used to collect data from reports, including how many reviewers collected data from each report, whether they worked independently, any processes for obtaining or confirming data from study investigators, and if applicable, details of automation tools used in the process.	Section 5.1 "Data Extraction" (lines 559-563): Data extraction followed PRISMA guidelines; key metrics (accuracy, sensitivity, specificity) and limitations tabulated; extraction performed by multiple authors with cross-verification
Data items	10a	List and define all outcomes for which data were sought. Specify whether all results that were compatible with each outcome domain in each study were sought (e.g. for all measures, time points, analyses), and if not, the methods used to decide which results to collect.	Section 4.4 & Tables 5-10: Outcomes included diagnostic accuracy, sensitivity, specificity, precision, recall, F1-score, AUC-ROC; primary performance metrics extracted as reported in original studies
	10b	List and define all other variables for which data were sought (e.g. participant and intervention characteristics, funding sources). Describe any assumptions made about any missing or unclear information.	Tables 5-10: Study characteristics included imaging modality, AI approach, dataset, reported results, strengths, and limitations; missing data noted as "Not reported" where applicable
Study risk of bias assessment	11	Specify the methods used to assess risk of bias in the included studies, including details of the tool(s) used, how many reviewers assessed each study and whether they worked independently, and if applicable, details of automation tools used in the process.	Tables 5-10 "Limitations" column; Section 6: Qualitative assessment of methodological limitations (dataset size, generalizability, interpretability); formal risk-of-bias tool not applied due to heterogeneity of AI study designs

Section and Topic	Item #	Checklist item	Location where item is reported
Effect measures	12	Specify for each outcome the effect measure(s) (e.g. risk ratio, mean difference) used in the synthesis or presentation of results.	Section 4.4 (lines 471-490) & Tables 5-10: Accuracy, precision, recall, F1-score, specificity, AUC-ROC reported as primary effect measures for classification performance
Synthesis methods	13a	Describe the processes used to decide which studies were eligible for each synthesis (e.g. tabulating the study intervention characteristics and comparing against the planned groups for each synthesis (item #5)).	Section 5.1 & Sections 5.2-5.7: Studies grouped by imaging modality (mammography, ultrasound, MRI, MBI, PET/SPECT, histopathology); eligibility confirmed against criteria in Section 5.1
	13b	Describe any methods required to prepare the data for presentation or synthesis, such as handling of missing summary statistics, or data conversions.	Section 5.1 "Data Extraction": Metrics extracted as reported; studies with incomplete performance data excluded or noted; no statistical conversions applied due to narrative synthesis approach
	13c	Describe any methods used to tabulate or visually display results of individual studies and syntheses.	Tables 5-10 (structured summary tables); Figures 1-8 (graphical visualizations, PRISMA flow diagram, publication timeline)
	13d	Describe any methods used to synthesize results and provide a rationale for the choice(s). If meta-analysis was performed, describe the model(s), method(s) to identify the presence and extent of statistical heterogeneity, and software package(s) used.	Sections 5.2-5.7: Narrative synthesis employed due to substantial heterogeneity in AI architectures, datasets, evaluation metrics, and imaging protocols across studies; meta-analysis not performed
	13e	Describe any methods used to explore possible causes of heterogeneity among study results (e.g. subgroup analysis, meta-regression).	Sections 5.2-5.7 & Section 6: Qualitative exploration of heterogeneity sources (dataset diversity, model architecture, preprocessing methods, clinical setting); subgroup organization by imaging modality
	13f	Describe any sensitivity analyses conducted to assess robustness of the synthesized results.	Not applicable: Narrative synthesis precluded formal sensitivity analysis; robustness discussed qualitatively in Section 6
Reporting bias assessment	14	Describe any methods used to assess risk of bias due to missing results in a synthesis (arising from reporting biases).	Not formally assessed: Narrative synthesis approach; potential publication bias acknowledged as limitation in Section 6
Certainty assessment	15	Describe any methods used to assess certainty (or confidence) in the body of evidence for an outcome.	Section 6 "Limitations": Qualitative assessment of evidence certainty considering study limitations, generalizability, and methodological heterogeneity; GRADE framework not applied due to review scope
**RESULTS**			
Study selection	16a	Describe the results of the search and selection process, from the number of records identified in the search to the number of studies included in the review, ideally using a flow diagram.	Figure 7 (PRISMA flow diagram); Section 5.1 (lines 556-558): 132 records identified → 65 studies included after screening and eligibility assessment
	16b	Cite studies that might appear to meet the inclusion criteria, but which were excluded, and explain why they were excluded.	Figure 7: Exclusion reasons documented—Retracted Studies (n=3), Biased Methodology (n=8), Wrong Setting (n=6); specific excluded studies available upon request
Study characteristics	17	Cite each included study and present its characteristics.	Tables 5-10: All 65 included studies cited with approach, results, strengths, and limitations; organized by imaging modality
Risk of bias in studies	18	Present assessments of risk of bias for each included study.	Tables 5-10 "Limitations" column; Section 6: Study-specific limitations summarized; overall methodological concerns discussed
Results of individual studies	19	For all outcomes, present, for each study: (a) summary statistics for each group (where appropriate) and (b) an effect estimate and its precision (e.g. confidence/credible interval), ideally using structured tables or plots.	Tables 5-10: Performance metrics (accuracy, sensitivity, specificity, AUC, etc.) reported for each study as presented in original publications
Results of	20a	For each synthesis, briefly summarise the characteristics and risk of	Sections 5.2-5.7: Modality-specific summaries of study characteristics, performance

Section and Topic	Item #	Checklist item	Location where item is reported
syntheses		bias among contributing studies.	ranges, and common limitations
	20b	Present results of all statistical syntheses conducted. If meta-analysis was done, present for each the summary estimate and its precision (e.g. confidence/credible interval) and measures of statistical heterogeneity. If comparing groups, describe the direction of the effect.	Sections 5.2-5.7: Narrative synthesis of performance trends by modality; no meta-analysis performed due to methodological heterogeneity
	20c	Present results of all investigations of possible causes of heterogeneity among study results.	Sections 5.2-5.7 & Section 6: Discussion of factors contributing to performance variability (dataset size, architecture choice, preprocessing, clinical context)
	20d	Present results of all sensitivity analyses conducted to assess the robustness of the synthesized results.	Not applicable: Narrative synthesis approach; robustness discussed qualitatively in Section 6
Reporting biases	21	Present assessments of risk of bias due to missing results (arising from reporting biases) for each synthesis assessed.	Section 6: Potential for publication bias and selective reporting acknowledged as limitation
Certainty of evidence	22	Present assessments of certainty (or confidence) in the body of evidence for each outcome assessed.	Section 6 "Limitations" & Section 7 "Conclusions": Qualitative confidence assessment; recommendations for future validation studies
**DISCUSSION**			
Discussion	23a	Provide a general interpretation of the results in the context of other evidence.	Section 7 (lines 1128-1137): Synthesis of AI advancements across modalities; comparison with prior reviews and clinical guidelines
	23b	Discuss any limitations of the evidence included in the review.	Section 6 (lines 1102-1127); Tables 5-10 "Limitations": Dataset heterogeneity, lack of prospective validation, computational constraints, interpretability challenges
	23c	Discuss any limitations of the review processes used.	Section 6: Narrative synthesis limits quantitative pooling; English-language restriction; potential for missed grey literature
	23d	Discuss implications of the results for practice, policy, and future research.	Section 7 (lines 1137-1150): Recommendations for standardization, explainability, multi-center validation, regulatory pathways, and equitable implementation
**OTHER INFORMATION**			
Registration and protocol	24a	Provide registration information for the review, including register name and registration number, or state that the review was not registered.	This systematic review was not registered in PROSPERO. Registration was not pursued due to the rapidly evolving nature of AI research and the focus on technological synthesis rather than clinical intervention outcomes.
	24b	Indicate where the review protocol can be accessed, or state that a protocol was not prepared.	A formal protocol was not prepared; however, the review methodology adhered to PRISMA 2020 guidelines as described in Section 5.1.
	24c	Describe and explain any amendments to information provided at registration or in the protocol.	Not applicable: Review not registered; no formal protocol
Support	25	Describe sources of financial or non-financial support for the review, and the role of the funders or sponsors in the review.	Declarations section (lines 1151-1153): "Funding: No funding was received."
Competing interests	26	Declare any competing interests of review authors.	Declarations section (lines 1151, 1156): "Conflict of Interest: No conflict of interest." / "Competing Interests: The authors declare no competing interests."
Availability of data, code and other materials	27	Report which of the following are publicly available and where they can be found: template data collection forms; data extracted from included studies; data used for all analyses; analytic code; any other materials used in the review.	The data extraction tables (Tables 5-10) and PRISMA checklist are included in this manuscript. Additional materials (search strategy details, excluded studies list) are available from the corresponding author upon reasonable request.

From: Page MJ, McKenzie JE, Bossuyt PM, Boutron I, Hoffmann TC, Mulrow CD, et al. The PRISMA 2020 statement: an updated guideline for reporting systematic reviews. BMJ 2021;372:n71. doi:

PRISMA 2020 Checklist

10.1136/bmj.n71
